# Are there jumps in evidence accumulation, and what, if anything, do they reflect psychologically? An analysis of Lévy Flights models of decision-making

**DOI:** 10.3758/s13423-023-02284-4

**Published:** 2023-07-19

**Authors:** Amir Hosein Hadian Rasanan, Jamal Amani Rad, David K. Sewell

**Affiliations:** 1 Institute for Cognitive and Brain Sciences, Shahid Beheshti University, Tehran, Iran; 2 Faculty of Psychology, University of Basel, Basel, Switzerland; 3 Department of Cognitive Modeling, Institute for Cognitive and Brain Sciences, Shahid Beheshti University, Tehran, Iran; 4 School of Psychology, The University of Queensland, St Lucia, QLD 4072 Brisbane, Australia

**Keywords:** Decision making, Evidence accumulation, Lévy Flights, Speed-accuracy tradeoff

## Abstract

According to existing theories of simple decision-making, decisions are initiated by continuously sampling and accumulating perceptual evidence until a threshold value has been reached. Many models, such as the diffusion decision model, assume a noisy accumulation process, described mathematically as a stochastic Wiener process with Gaussian distributed noise. Recently, an alternative account of decision-making has been proposed in the Lévy Flights (LF) model, in which accumulation noise is characterized by a heavy-tailed power-law distribution, controlled by a parameter, $\alpha$. The LF model produces sudden large “jumps" in evidence accumulation that are not produced by the standard Wiener diffusion model, which some have argued provide better fits to data. It remains unclear, however, whether jumps in evidence accumulation have any real psychological meaning. Here, we investigate the conjecture by Voss et al. (*Psychonomic Bulletin & Review,* *26*(3), 813–832, 2019) that jumps might reflect sudden shifts in the source of evidence people rely on to make decisions. We reason that if jumps are psychologically real, we should observe systematic reductions in jumps as people become more practiced with a task (i.e., as people converge on a stable decision strategy with experience). We fitted five versions of the LF model to behavioral data from a study by Evans and Brown (*Psychonomic Bulletin & Review*, *24*(2), 597–606, 2017), using a five-layer deep inference neural network for parameter estimation. The analysis revealed systematic reductions in jumps as a function of practice, such that the LF model more closely approximated the standard Wiener model over time. This trend could not be attributed to other sources of parameter variability, speaking against the possibility of trade-offs with other model parameters. Our analysis suggests that jumps in the LF model might be capturing strategy instability exhibited by relatively inexperienced observers early on in task performance. We conclude that further investigation of a potential psychological interpretation of jumps in evidence accumulation is warranted.

**Supplementary Information:**

The online version contains supplementary material available at 10.3758/s13423-023-02284-4.

Theories of decision-making are fundamental to our understanding of psychology, neuroscience, and neuroeconomics, and have been developed and systematically tested over the last 60 years (Evans & Wagenmakers, 2020; Ratcliff et al., 2016; Sewell & Smith, 2016). The dominant view that has emerged over this time is that simple decisions can be conceptualized as a noisy evidence accumulation process, and mathematically represented by the class of sequential sampling models (SSMs; Ratcliff & Smith, 2004; Stone, 1960). In these models, stimulus information is continuously sampled, with each sample contributing a quantity of evidence favoring one or more response alternatives. The evidence from successive samples is summed, or accumulated through time until a criterion amount of evidence for one response alternative is accrued, initiating a behavioral response for that alternative. The success of this theoretical framework is reflected in the breadth of domains the models have been applied to, such as evaluating the optimality of decision policies (Bogacz et al., 2006; Drugowitsch et al., 2012; Evans et al., 2020; Evans & Brown, 2017; Evans et al., 2018; Starns & Ratcliff, 2012), stop signal paradigms (Matzke et al., 2013, 2017a, b), Go/No-Go paradigms (Gomez et al., 2007; Ratcliff et al., 2018), multi-attribute and many-alternatives choice (Kvam, 2019; Mallahi-Karai & Diederich, 2019, 2021; Roe et al., 2001; Trueblood et al., 2014; Usher & McClelland, 2004), learning strategies (Fontanesi et al., 2019; Miletic et al., 2021; Pedersen et al., 2017; Sewell et al., 2019; Sewell & Stallman, 2020), attentional choice (Gluth et al., 2020; Krajbich et al., 2010, 2012), continuous responses (Ratcliff, 2018; Smith, 2016), neural processes (Gold & Shadlen, 2007), and so on.

The most popular and well-studied model of simple decision-making is the diffusion decision model (DDM) of Ratcliff (Ratcliff, 1978; Ratcliff & McKoon, 2008; Ratcliff & Rouder, 1998). The DDM represents evidence as a single signed value, *X*(*t*), that is accumulated from a starting point, *z*, toward one of two absorbing boundaries, located at zero and *a*. Psychologically, the boundary separation parameter, *a*, characterizes response caution, with more widely separated boundaries reflecting more cautious responding (i.e., a greater quantity of evidence is required to trigger a response). The start-point parameter, *z*, is interpreted psychologically as characterizing response bias, and varies uniformly with mean *z* and range $s_{zr}$. An unbiased decision-maker will begin evidence accumulation from $z=\frac{a}{2}$. For a biased decision-maker, the accumulation process will begin closer to the boundary favored by the bias. The rate of evidence accumulation, the drift rate of the diffusion process, has a mean *v* and standard deviation $s_{v}$ across trials. Psychologically, the drift rate is determined by the quality of the stimulus. The sign of the drift rate determines which response alternative evidence tends to accumulate towards. Drift rates with absolute values that deviate further from zero correspond to stimuli with higher-quality information that more readily discriminates between response alternatives. Mathematically, evidence accumulation in the DDM can be described as,

1 $$\begin{aligned} X(t+\Delta t) = X(t) + v \cdot \Delta t + \mathcal {N}(0,1) \cdot \sqrt{\Delta t}, \end{aligned}$$

where $\mathcal {N}$ denotes a standard normal distribution and $\Delta t$ represents the time step. The accumulation dynamics of Eq.  1 determine the decision time, but behavioral response times also consist of the time required to encode the stimulus and execute a response. In the DDM, these components of non-decision time are described by the parameter, $t_0$ (also written as $T_{er}$). Non-decision time is assumed to vary uniformly with mean $t_0$ and range $s_{t}$. Components of the DDM (i.e., without variability parameters) are illustrated in Fig. 1.

**Fig. 1:**
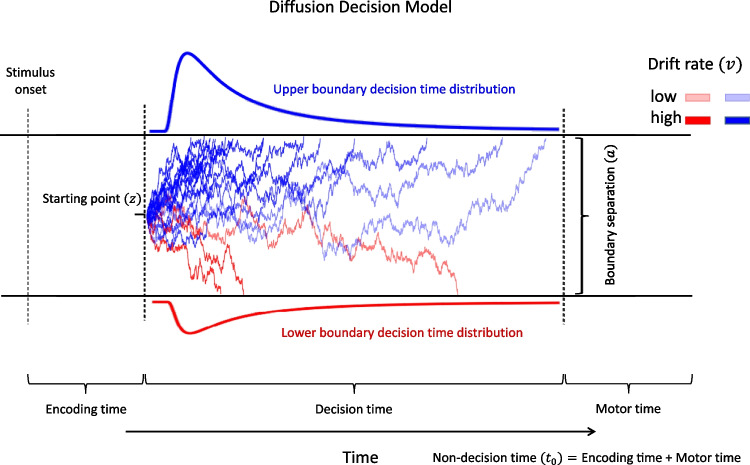
A schematic view of the diffusion decision model. The jagged lines depict evidence accumulation trajectories driven by different stimuli across different trials. The darker lines correspond to higher-quality stimuli that produce drift rates that deviate further from zero. The blue and red trajectories were produced by different stimuli (e.g., rightward vs. leftward motion), which drive the accumulation process toward different absorbing boundaries. The reader is referred to the online version of this article for a colored version of the figure

A number of alternatives to the DDM have been developed. These include “reduced" forms of the standard DDM, incorporating simplifying assumptions that remove between-trial parameter variability (Wagenmakers et al., 2007), as well as models that make different assumptions about how evidence is represented in the model. For example, multi-accumulator models (e.g., Brown & Heathcote, 2005, 2008; Hawkins & Heathcote, 2021; Smith, 2022; Smith & Vickers, 1988; Tillman et al., 2020; Usher & McClelland, 2001), represent separate absolute evidence totals for each response alternative that race against each other, rather than a single signed relative evidence total. More recently, Voss and colleagues (Voss et al., 2019; Wieschen et al., 2020) have proposed the Lévy Flights (LF) model as another alternative, which includes the DDM as a special case. The LF model differs from the DDM in assuming that there are random “jumps" in evidence accumulation that do not conform to the standard Gaussian noise process described in Eq. 1. To allow for these large sudden changes in evidence accumulation, the noise in the accumulation process is instead characterized by a heavy-tailed $\alpha$-stable distribution with long-tailed, power-law asymptote $\lambda (x)\sim |x|^{-1-\alpha }$ ($0<\alpha \le 2$) (Padash et al., 2019). Evidence accumulation in the LF model can therefore be written as,

2 $$\begin{aligned} X(t+\Delta t)= &  X(t) + v \cdot \Delta t + Stable(\alpha ,\beta =0,\gamma \nonumber \\= &  \frac{1}{\sqrt{2}},\delta =0) \cdot \Delta t^{\frac{1}{\alpha }}. \end{aligned}$$

In Eq. 2, $\alpha$, $\beta$, $\gamma$, and $\delta$ are the parameters of an $\alpha$-stable distribution, where $\alpha \in (0,2]$ is the stability index (Lévy index), $\beta \in [-1,1]$ is the skewness parameter, $\gamma >0$ is the scale parameter, and $\delta$ is the shift parameter that can be any real number. In the LF model, the distribution of accumulation noise is $\alpha$-stable with fixed parameters $\beta = 0$, $\gamma = \frac{1}{\sqrt{2}}$, and $\delta = 0$ (Gikhman & Skorokhod, 1975; Samorodnitsky & Taqqu, 1994) and $\alpha$ is the free parameter which is constrained to $1\le \alpha \le 2$ (i.e. $p^{\alpha } (x) = Stable(\alpha , \beta =0, \gamma = \frac{1}{\sqrt{2}}, \delta = 0)$, and $1\le \alpha \le 2$). When $\alpha =2$, the accumulation noise is Gaussian, and the model is equivalent to the DDM (Eq. 1). When $\alpha =1$, the accumulation noise is Cauchy distributed. Accumulation dynamics for the standard DDM and the LF model with different $\alpha$ values are shown in Fig. 2.

**Fig. 2:**
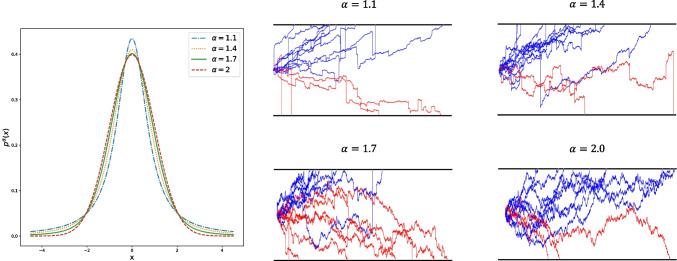
Plot of sample paths for the Lévy Flights model for different values of $\alpha$. In the left panel, the noise distribution of the accumulation process is presented for different values of $\alpha$. The right panels show sample accumulation paths under different values of $\alpha$. When $\alpha =2$, accumulation noise is Gaussian, and the model is equivalent to the standard diffusion decision model. As $\alpha$ takes on values lower than 2, accumulation noise becomes more variable, and large jumps in the accumulation process, depicted as sudden spikes in the accumulation paths, become more commonplace. The reader is referred to the online version of this article for a colored version of the figure

To date, the LF model has only been applied sparingly in psychological research. The domains in which the model has proved useful, however, are quite broad, having been applied to memory search and retrieval processes (Patten et al., 2020), semantic memory search processes (Montez et al., 2015; Rhodes & Turvey, 2007), spatial memory (Kerster et al., 2016), perception in typically developing children and children with autism spectrum disorder (Liberati et al., 2017), and animal foraging (Reynolds, 2012). There are also neurological motivations for considering the LF model as a model of decision-making. For example, Wardak and Gong (2021), introduced a dynamical model for spiking neurons in heterogeneous networks based on LF. Moreover, Liu et al. (2021) have shown that LF can explain oscillatory activity in primate cerebral cortex.. As mentioned above, Voss and colleagues have recently used the LF model as a model of decision-making (Voss et al., 2019; Wieschen et al., 2020), arguing for several potential benefits over the DDM, which we now summarize.

First, Voss et al. (2019) showed that LF models can provide highly accurate accounts of fast error patterns in data. In the DDM, fast errors have been previously explained in terms of start-point variability (Ratcliff & Rouder, 1998; Smith et al., 2014). While start-point variability has good psychological plausibility–it can be related theoretically to sequential effects in stimulus presentation and/or responding (e.g., Rabbitt, 1969), an account that has received support from cognitive modeling (e.g., Bode et al., 2012)–reliance on between-trial variability parameters has been criticized due to relatively poor parameter recovery properties (Lerche & Voss, 2016; Voss et al., 2019). The LF model can account for errors that are, on average, faster than correct responses without between-trial variability in start-point, attributing them to large jumps that occur early in evidence accumulation that quickly move the process toward the incorrect absorbing boundary. This account can be investigated in more detail by examining correlations between the $\alpha$ parameter and overall error rate (Wieschen et al., 2020).

Second, extending the applications to data exhibiting fast errors, the LF model may be useful for examining time-pressured decisions, where fast decisions are more important than accurate ones. In these paradigms, a decision-maker can choose among three types of strategies: (1) maintaining a low fixed evidence threshold, (2) implementing a collapsing evidence threshold (Cisek et al., 2009; Ditterich, 2006; Drugowitsch et al., 2012; Hawkins et al., 2015; Thura et al., 2012; Zhang et al., 2014), or (3) capitalizing on sudden jumps during evidence accumulation. A low fixed evidence threshold leads to many incorrect decisions in difficult conditions and is therefore not a good option (Evans et al., 2020). A collapsing threshold overcomes the problem of an unacceptably high error rate in difficult conditions, but does not produce fast errors (Evans et al., 2020). Taking advantage of sudden jumps in evidence allows the participant to reach an acceptable level of overall accuracy in this type of time-pressured environment while also accounting for fast errors. Wieschen et al. (2020) found high positive correlations between response time (RT) and the $\alpha$ parameter, as well as between overall accuracy and the $\alpha$ parameter: the shorter the response time (or the faster the response), the smaller the $\alpha$ parameter, which implies that sudden jumps in evidence accumulation contributed to these faster decisions.

Third, jumps in evidence accumulation can be related to the “jumping to a conclusion" phenomenon (McKay et al., 2006), which is not readily achieved within a DDM framework. The heavy tails in the accumulation process outlined by the LF model are controlled by the value of the $\alpha$ parameter. Psychologically, lower values of $\alpha$ could correspond to a higher probability of suddenly accumulating an extreme amount of evidence (Wieschen et al., 2020). This phenomenon may occur in groups with high impulsivity, whose decision strategies may be more naturally modeled as a LF process due to lack of stability. Sudden accumulation of evidence might also be interpretable as a consequence of changes in the allocation of attentional resources that arise through the use of a hypothesis testing strategy (e.g., toggling between different decision strategies when judging a multi-attribute stimulus; cf. Lamberts, 1995).

Notwithstanding the potential advantages of using the LF model as a generalized form of the DDM, it is unclear whether situations where the LF outperforms the DDM are best attributed to the LF model providing a more accurate characterization of within-trial noise, or simply to increased model flexibility. Although Voss and colleagues have argued for the former (Voss et al., 2019; Wieschen et al., 2020), support for the LF model as a decision-making model has come primarily from assessing goodness of fit against other competing models (e.g., the standard DDM and various collapsing threshold models), and not from a more detailed theoretical analysis of the critical $\alpha$ parameter.

We address this issue by exploring Voss et al.’s (2019) conjecture that jumps in evidence accumulation may be due to sudden on-the-fly shifts of attention that may reflect instability in the decision strategy (or the capacity to execute the strategy) adopted by the observer. We argue that if the jumps in evidence accumulation assumed by the LF model can be psychologically interpreted in this way, we should be able to observe systematic changes in $\alpha$, as people become more experienced in performing a task or become more familiar with the stimuli they are presented with. That is, the prevalence of sudden jumps in evidence accumulation should progressively reduce as people settle on a consistent decision strategy for performing a task and/or grow more adept at parsing and encoding stimuli in a way that supports effective task performance. This means that people’s early decision performance might best be modeled using an $\alpha$ value that is less than 2, but with increasing experience over the course of an experiment, approaches a value of 2, approximating the behavior of the standard DDM. If, on the other hand, $\alpha$ cannot be psychologically interpreted in this way–if it enables better fits to data by simply increasing flexibility–we should not observe any systematic changes in the parameter with experience. To further explore the issue of model flexibility, we also consider whether the $\alpha$ parameter trades off with either mean drift rate or other between-trial variability parameters in the standard DDM (e.g., variability in start-point).

We structure the rest of the article as follows. We first provide a more detailed mathematical overview of the LF model. We then report a re-analysis of data from Evans and Brown (2017) that enables tracking of the $\alpha$ parameter (and other LF model parameters) as a function of task experience. In their study, Evans and Brown (2017) analyzed people’s performance in a motion discrimination task using the DDM, showing that people set overly cautious decision thresholds at the beginning of the task, but relax their thresholds with experience in a way that approaches an optimal setting (i.e., one that maximizes the rate of reward on the task). Since reductions in decision threshold over time reflect systematic changes in the way people approach the task—how the quality of information extracted from the stimulus is reconciled with the need to respond quickly and accurately—one might predict that if the $\alpha$ parameter reflects instability in executing a decision strategy (or shifting attention among multiple competing strategies), its value should systematically change in a way that reflects the gradual refinement of (or convergence toward) a stable encoding and decision strategy. Comparing changes in $\alpha$ over time with corresponding changes in other between-trial variability parameters (i.e., $s_{v}$, $s_{zr}$, and $s_{t}$) allows us to judge whether $\alpha$ is simply acting as a surrogate for one of these parameters, which would suggest that improvements in fit afforded by the LF model might reflect greater model flexibility rather than a more accurate description of psychological processing.

## Lévy Flights Model

Mathematically, the Lévy Flights model is a generalized continuous-time random walk process, a sequential sampling model (Voss et al., 2019) that uses an $\alpha$-stable jump length distribution (or Lévy distribution Gnedenko & Kolmogorov, 1954) with a long-tailed, power-law asymptote $\lambda (x)\sim |x|^{-1-\alpha }$ ($0<\alpha \le 2$) to describe noise in the accumulation process (Hadian Rasanan et al., 2022; Padash et al., 2019, 2020). Therefore, information accumulation in LF model can be formulated as follows:

3 $$\begin{aligned} {\left\{ \begin{array}{ll} X(0) = z,\\ X(t+\Delta t) = X(t) + v\cdot \Delta t + e \Delta t^{\frac{1}{\alpha }}, ~~~ e \sim p^{\alpha }(x), ~~~1<\alpha <2, \end{array}\right. }, \end{aligned}$$

where $p^{\alpha } (x) = Stable(\alpha , \beta =0, \gamma = \frac{1}{\sqrt{2}}, \delta =0)$, *v* is the drift rate, and the process terminates whenever $X(t)\ge a$ or $X(t) \le 0$. A technical point concerns the different ranges of values $\alpha$ can take with respect to psychological plausibility. The value of the $\alpha$ parameter ranges between 0 to 2 in the $\alpha$-stable distribution. However, when $\alpha$ is lower than 1, the resulting accumulation process becomes an anomalous sub-diffusion process that has not been considered in the decision-making literature. In keeping with theoretical precedent within psychology, we restrict the range of $\alpha$ in Eq. 3 to lie between 1 and 2. By considering Eq. 3, the process can be considered as a continuous time process $vt + Z_{t}$, in which:

4 $$\begin{aligned} Z_t = \frac{e_1 + \cdots + e_{ct}}{ct^{\frac{1}{\alpha }}}. \end{aligned}$$

Thus, the Fourier Transform of the probability density function of the location of the accumulated evidence total is obtained as follows:

5 $$\begin{aligned} \hat{p}(k, t) =E[e^{-ik(vt + Z_{t})}] = e^{-ikvt + r(ik)^{\alpha } + q(-ik)^{\alpha }}, \end{aligned}$$

where *r* represents the probability of jumping upward, and *q* is the probability of jumping downward. Moreover, it can be concluded that the $\hat{p}(k, t)$ satisfies the following differential equation (Meerschaert & Sikorskii, 2011):

6 $$\begin{aligned} \frac{d \hat{p}(x, t)}{dt} = - i k v + r (ik^{\alpha }) + q (-ik^{\alpha }). \end{aligned}$$

By applying the inverse Fourier Transform on both sides of Eq. 6 the following space fractional partial differential equation is obtained (Meerschaert & Sikorskii, 2011; Padash et al., 2019; Hadian Rasanan et al., 2022):

7 $$\begin{aligned} \frac{\partial }{\partial t} p(x, t) + v \frac{\partial }{\partial x} p(x, t) = D_{x}^{\alpha } p(x, t), \end{aligned}$$

and by regarding the starting and termination conditions of the process, the following initial and boundary conditions are obtained:

8 $$\begin{aligned} p(x, 0) = \delta (x-z), ~~~~~p(a, t) = p(0, t) = 0. \end{aligned}$$

The fraction derivative in Eq. 7 (i.e. $D^{\alpha }_{x}$) is defined as follows:

9 $$\begin{aligned} D_{x}^{\alpha } p(x, t) =\frac{-1}{2 \cos {\frac{\alpha \pi }{2}}} \Big ({ ^{R}_{-\infty }D_x^{\alpha }} p(x, t) + _{x}^{R}D_{\infty }^{\alpha } p(x, t)\Big ), \end{aligned}$$

where $_{-\infty }^{R}D_{x}^{\alpha }$ and ${ ^{R}_{x}D_{\infty }^{\alpha }}$ are the left and right Riemann-Liouville fractional derivatives and have the following definitions (Ding & Li, 2017; Hadian Rasanan et al., 2020):

10 $$\begin{aligned} _{-\infty }^{R}D_{x}^{\alpha } p(x, t) = \frac{1}{\Gamma (2-\alpha )}\frac{d^2}{dx^2}\int _{-\infty }^{x} \frac{p(\xi , t)}{(x-\xi )^{\alpha -1} } d\xi , \end{aligned}$$

11 $$\begin{aligned} ^{R}_{x}D_{\infty }^{\alpha } p(x, t) = \frac{1}{\Gamma (2-\alpha )}\frac{d^2}{dx^2}\int _{x}^{\infty } \frac{p(\xi , t)}{(\xi - x)^{\alpha -1} } d\xi . \end{aligned}$$

By considering Eq. 7, the probability of the location of the accumulated evidence total at time *t* can be obtained by solving the space fractional partial differential equation. Thus, by approximating the solution of this equation the survival probability of the decision process can be obtained as:

12 $$\begin{aligned} S(t) = \int _{0}^{a} p(x, t) dx, \end{aligned}$$

which determines whether the location of the accumulated evidence total is still somewhere between the absorbing boundaries at time *t*, or if the process has already terminated at one of them.

## Evans & Brown (2017) Data Reanalysis

We now present a reanalysis of data from a study by Evans and Brown (2017), using the LF model. Their study examined reward rate optimality in perceptual decision-making when people had either a fixed amount of time to complete the task, or a fixed number of trials to complete. This data set was selected because it allows us to examine whether the $\alpha$ parameter is sensitive to refinement in people’s decision-making strategy over time. The task also varied the level of performance feedback provided to participants, providing more fine-grained information on factors that influence the rate at which people’s response strategy becomes tuned to the task. For simplicity, our analysis focuses on the fixed-trial conditions from their study, in which participants completed a fixed number of trials in a motion discrimination task using random dot kinematogram stimuli (Roitman & Shadlen, 2002). On each trial, participants viewed an array of moving dots, a proportion of which moved coherently in one direction. Participants were randomly assigned to one of three conditions that differed in terms of the level of feedback provided at the end of each trial block. In the ‘low information’ condition, participants were only informed that the block had been completed. In the ‘medium information’ condition, participants were informed about their performance in that block (i.e., how many points they accrued in the block, the time taken to complete the block, and the rate at which they accrued points). In the ‘high information’ condition, some hints on how participants could improve their performance were presented in addition to the summary provided in the medium information condition (i.e., participants were advised that responding faster/slower could adjust their performance in a way that increased the rate at which they accrued points). The key finding from the study was that people tend to perform non-optimally by being overly cautious. However, with highly informative feedback and practice on the task, people’s performance rapidly approaches optimality.

### Fits of the Lévy flights model to human data

There were 85 total participants in the original data set, of whom 39 were assigned to one of the fixed-trial conditions that we focus on. Following Evans and Brown (2017), we removed nine participants with accuracy less than 70%, yielding 30 participants with data that we modeled (i.e., 10 participants in the ‘low information’ condition, 9 in the ‘medium information’ condition, and 11 in the ‘high information’ condition). Evans and Brown analyzed the data from each of the 24 blocks of trials using the DDM, yielding parameter estimates for each individual block. For our analysis, the relatively low number of trials per block (N = 40) presented difficulties in parameter recovery for the LF model, and so we elected to collapse the data into four larger trial epochs. Each epoch consisted of six blocks of the original design (e.g., Epoch 1 consisted of data from trial blocks 1-6 of the Evans and Brown study). Each epoch in our analysis comprised N = 240 trials.

Our main interest was to examine changes in the LF jump parameter, $\alpha$, over the course of the task. If this parameter reflects instability in selecting or executing a decision strategy, as conjectured by Voss et al. (2019), we would expect to see systematic changes in $\alpha$ in the practice data of Evans and Brown (2017). To this end, we compared five versions of the LF model, each instantiating different assumptions about the latent processes involved in decision making. If people’s stability in strategy selection or execution increases with practice, we would expect a model that allows $\alpha$ to vary over time to successfully account for the data while also showing reductions in the rate of jumps in evidence accumulation. If, on the other hand, $\alpha$ serves to simply increase the flexibility of the model—improving fit, but without demonstrating any clear systematic mapping with performance or task parameters—we would not expect to see any clear changes in $\alpha$ as people become more experienced with the task. We can further consider whether $\alpha$ adds flexibility by duplicating the effects of other DDM parameters that describe across-trial variability in different processing components. Specifically, we investigate whether the effects of allowing $\alpha$ to vary over time can be captured by across-trial variability in drift rate, the start-point of evidence accumulation, and non-decision time. Finally, because refinement of a decision strategy might be described more simply in terms of increases in drift rate—a product of improved stimulus encoding potentially driven by selective attention mechanisms more effectively facilitating focus on the most task-relevant properties of the stimulus—we also consider whether the effects of $\alpha$ can be explained in terms of changes in mean drift rate.

**Table 1:** Summary of free parameters for the five models fitted to preregistered data by Evans and Brown (2017)

Model	Free parameters	# Free parameters
$\alpha$ Varying Model	$\{v, t_{0}, z, a_{1}, a_{2}, a_{3}, a_{4}, \alpha _{1}, \alpha _{2}, \alpha _{3}, \alpha _{4}\}$	11
Drift Variability Model	$\{v, z, t_{0}, \alpha , a_{1}, a_{2}, a_{3}, a_{4}, sv_{1}, sv_{2}, sv_{3}, sv_{4}\}$	12
Start-Point Variability Model	$\{v, t_{0}, z, \alpha , a_{1}, a_{2}, a_{3}, a_{4}, szr_{1}, szr_{2}, szr_{3}, szr_{4}\}$	12
Non-Decision Time Variability Model	$\{v, t_{0}, z, \alpha , a_{1}, a_{2}, a_{3}, a_{4}, st_1, st_{2}, st_{3}, st_{4}\}$	12
Drift & $\alpha$ Varying Model	$\{t_{0}, z, a_{1}, a_{2}, a_{3}, a_{4}, v_{1}, v_{2}, v_{3}, v_{4}, \alpha _{1}, \alpha _{2}, \alpha _{3}, \alpha _{4}\}$	14

**Fig. 3:**
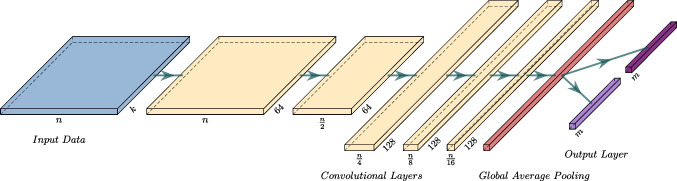
The architecture of the Deep Inference neural network we used for parameter estimation in which *m* denotes the number of parameters that the network is estimating, *k* denotes the number of epochs, and *n* denotes the size of the input data

The models were fitted to the data from the high, medium, and low information conditions such that all model parameters were allowed to vary across conditions (i.e., different parameter estimates across different conditions). For each model, we estimated a decision threshold for each trial epoch ($a_{1}$, $a_{2}$, $a_{3}$, $a_{4}$). Following Evans and Brown (2017), we also estimated a single drift rate (*v*) for the entire experiment in the first four models. To examine whether their practice effects can be captured by the drift rate parameter we estimated four drift rates alongside epoch-wise estimates of $\alpha$ in the fifth model. For all models, we estimated a single relative starting point bias ($z_{r} = \frac{z}{a}$), and a single non-decision time ($t_{0}$) for the entire experiment. In the model of primary interest, the $\alpha$ *varying model*, we estimated a separate $\alpha$ parameter for each trial epoch. We did not estimate any sources of between-trial parameter variability for this model (i.e., $s_{v}$, $s_{zr}$, and $s_{t}$ were all fixed to 0). We also considered several control models that all assumed a fixed level of $\alpha$ across the experiment, but allowed a different between-trial variability parameter to differ across trial epochs. The rationale for the control models was to determine whether the jump parameter in the LF model is simply mimicking the effects of one of the between-trial variability parameters, which confer some additional flexibility to the standard DDM. Specifically, we fitted three such control models, the *drift rate variability model*, the *start-point variability model*, and *non-decision time variability model*, which each allowed their namesake parameter, $s_{v}$, $s_{zr}$, or $s_{t}$, respectively to vary across trial epochs. We also fitted another control model, the *drift &* $\alpha$ *varying* model, in which both mean drift rate and $\alpha$ parameters were free to vary across trial epochs. To the extent that any systematic changes in $\alpha$ are mirrored in the other between-trial variability parameters or drift rate, we can conclude that $\alpha$ primarily contributes flexibility to the LF model without having a clear psychological interpretation that is distinct from existing model parameters. Table 1 summarizes the free parameters for the five models fitted to the data.

While there have been recent attempts for approximating the likelihood function the LF model using partial differential equations (Hadian Rasanan et al., 2022), the lack of a closed-form likelihood function typically impedes Bayesian inference methods (Fengler et al., 2021; Voss et al., 2019). A notable paradigm of approximating the likelihood function is approximate Bayesian computing (ABC; Turner & Van Zandt, 2012). There are various ABC algorithms for likelihood approximation, but recently, some researchers have combined neural network approaches with traditional ABC algorithms and have improved them (Fengler et al., 2021; Radev et al., 2020). Previously, a convolutional neural network (CNN) was developed by Radev et al. (2020) to approximate the likelihood function. This network can approximate the parameters of a stochastic process by learning a large number of simulations of that process. We fit the four variants of the LF models described above using a deep inference algorithm based on the CNN described by Radev et al. (2020). This neural network approach learns approximate likelihoods for the LF models, allowing fast posterior sampling with only a one-off cost for model simulations that are amortized for future inference. Parameter estimation for each of the five LF models was achieved by training its own dedicated CNN. For example, for the *non-decision time variability model*, we generated 28000 data-sets with 240 trials of the LF model, and the network is trained using these data-sets. Each network had 5 layers and the size of filters in each layer were 64, 64, 128, 128, and 128, respectively. Additionally, each network consisted of one channel for each trial epoch (i.e., four channels in total). The architecture of the utilized network is presented in Fig. 3. For the $\alpha$ varying model, the network estimates a drift rate, a non-decision time, a bias for the experiment, and four $\alpha$ and four decision threshold parameters (one for each trial epoch).

**Fig. 4:**
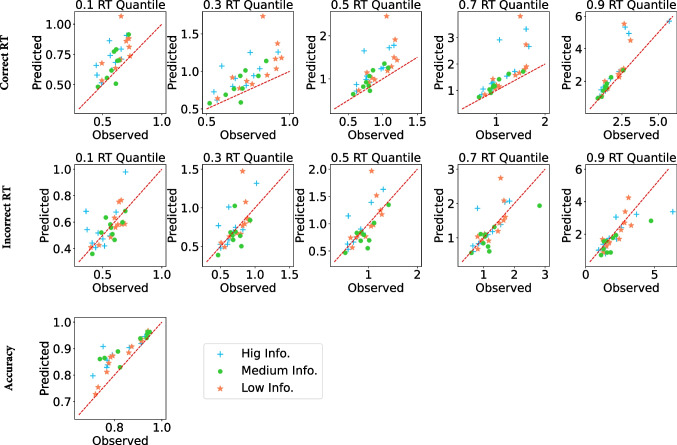
Predictions of the $\alpha$ *varying model* against observed data in different conditions for correct RTs in seconds (top panels), error RT (middle panels), and accuracy (bottom panel). Across columns, panels in the top two rows show response times for the 0.1, 0.3, 0.5, 0.7, and 0.9 distribution quantiles. Each point shows data for one participant

### Modeling results

We now report the results of fitting the LF models to the individual data from each condition of the Evans and Brown (2017) study. We report summaries of the parameter estimates in the main text, and more detailed information about parameter estimates at the individual level in the Supplementary Materials.

We first consider the quality of fit for the $\alpha$ *varying model* to the data from the three information conditions. Figure 4 plots model predictions against observed data for the RT distribution data for correct responses (top panels), error responses (middle panels), and accuracy (bottom panel). The model provides a reasonable account of the data, though there is a general tendency for the model to predict slower correct RTs than are observed empirically, and to overestimate accuracy. The overestimation of the fastest correct RTs and accuracy appears most pronounced for the high information condition.

**Fig. 5:**
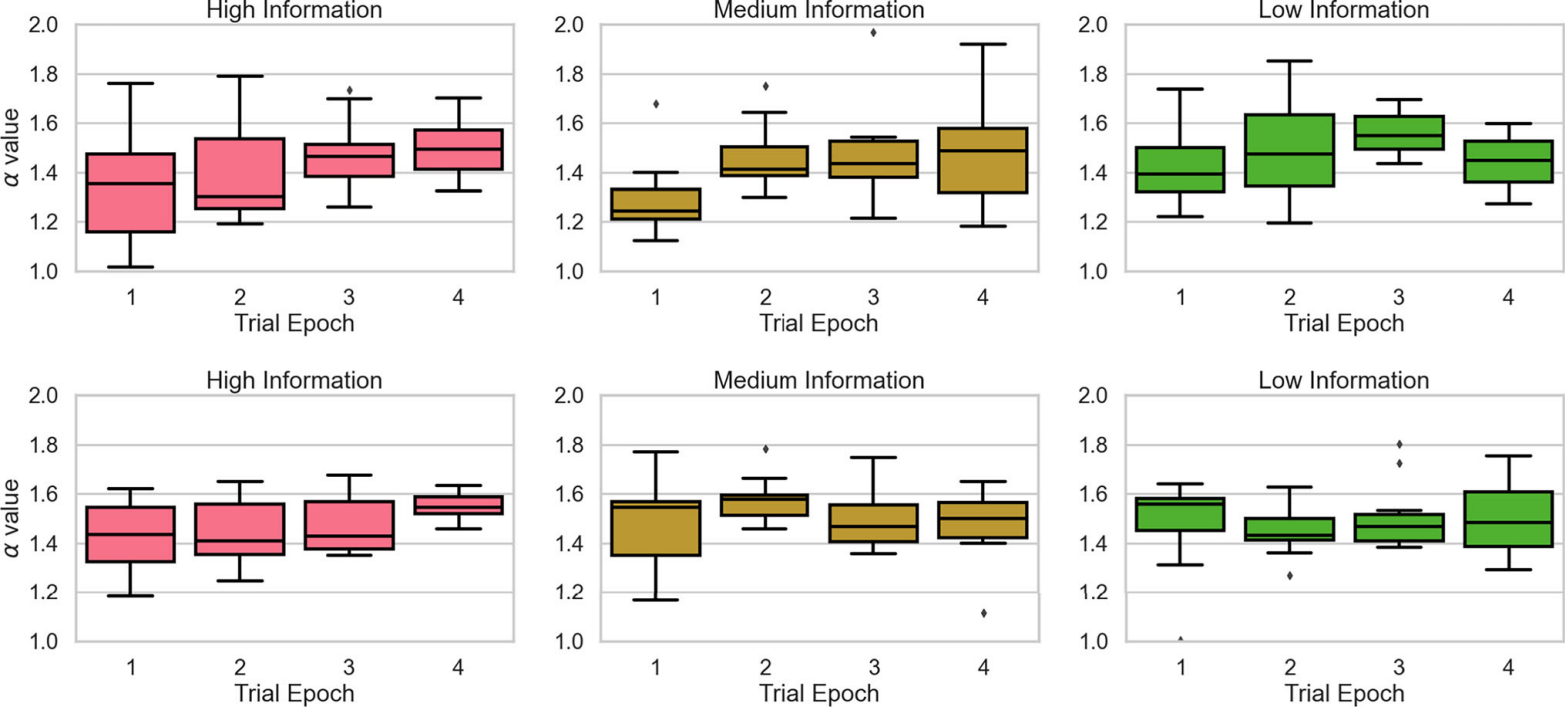
Box plots depicting changes in the $\alpha$ parameter across trial epochs for the $\alpha$ *varying model* (top panels) and $\alpha$ *& drift varying model* (bottom panels) in the High (left panels), Medium (middle panels), and Low Information (right panels) conditions. For the $\alpha$ *varying model* model, averaged across participants, the $\alpha$ parameter increases by 17%, 17.5%, and 2.2% in the last trial epoch relative to the first, for the High, Medium, and Low information conditions respectively. Similarly, for the $\alpha$ *& drift varying model*, averaged across participants, $\alpha$ in the final trial epoch is increased by 9.7%, 0.5%, and 3.5% relative to the first, for the High, Medium, and Low information conditions respectively

**Table 2:** Fitting performance of all models. The best performing model for each fit index (log likelihood, AIC, and BIC) is shown in bold

	Model 1	Model 2	Model 3	Model 4	Model 5
log likelihood	**-61624**	-70157	-90672	-164413	-68658
AIC	**123578**	140674	181704	329187	137737
BIC	**125514**	142786	183817	331299	140201

*Note*. Model 1 = $\alpha$ Varying Model; Model 2 = Drift Variability Model; Model 3 = Start-Point Variability Model; Model 4 = Non-Decision Time Variability Model; Model 5 = Drift & $\alpha$ Varying Model

**Fig. 6:**
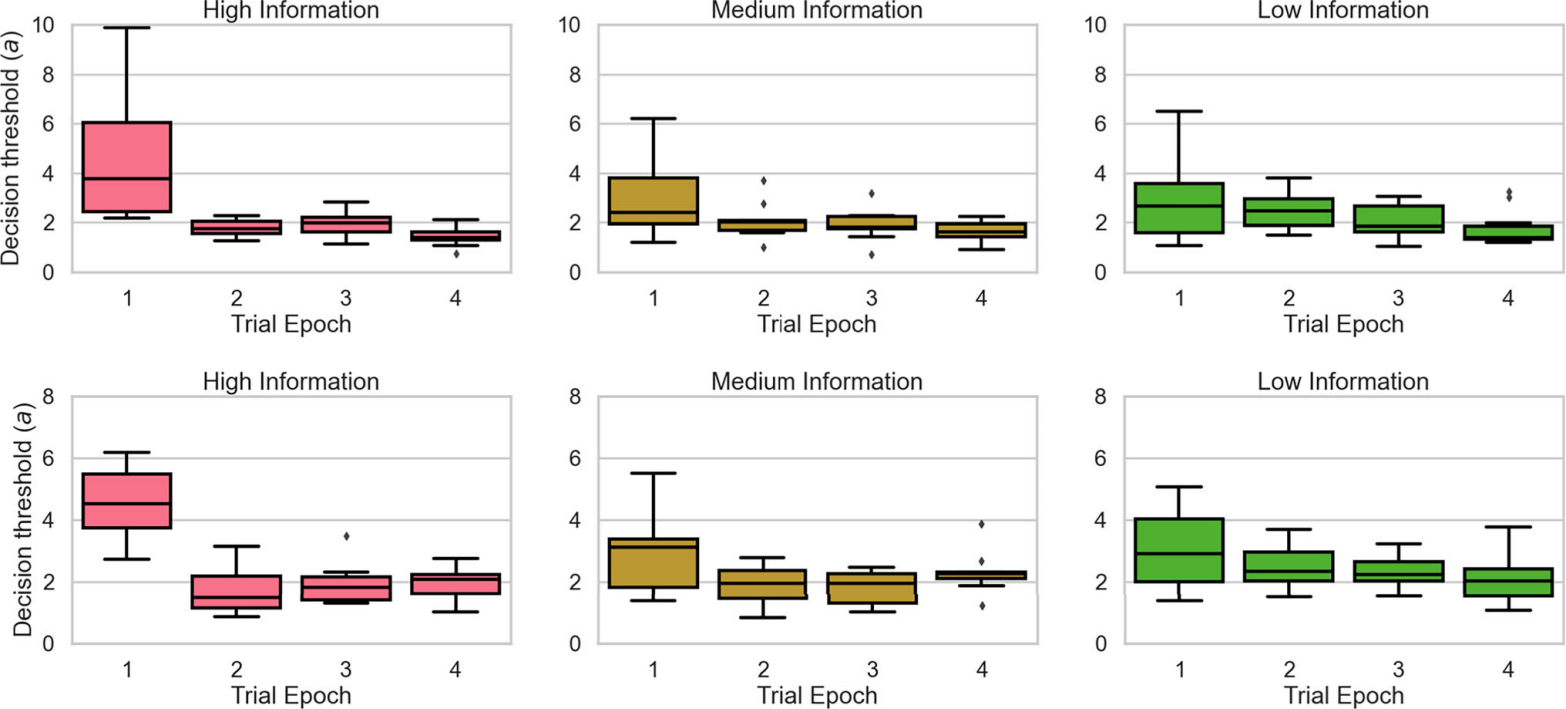
Box plots depicting changes in the decision threshold (*a*) parameter across trial epochs for the $\alpha$ *varying model* (top panels) and the *drift and *$\alpha$ *varying model* (bottom panels) in the High (left panel), Medium (middle panel), and Low Information (right panel) conditions. For the $\alpha$ *varying model* model, averaged across participants, the decision threshold decreases by 60.7%, 33.4%, and 20.7% in the last trial epoch relative to the first, for the High, Medium, and Low information conditions respectively. Similarly, for the $\alpha$ *& drift varying model*, averaged across participants, the decision threshold in the final trial epoch is reduced by 52%, 4.5%, and 16.5% relative to the first, for the High, Medium, and Low information conditions respectively

**Fig. 7:**
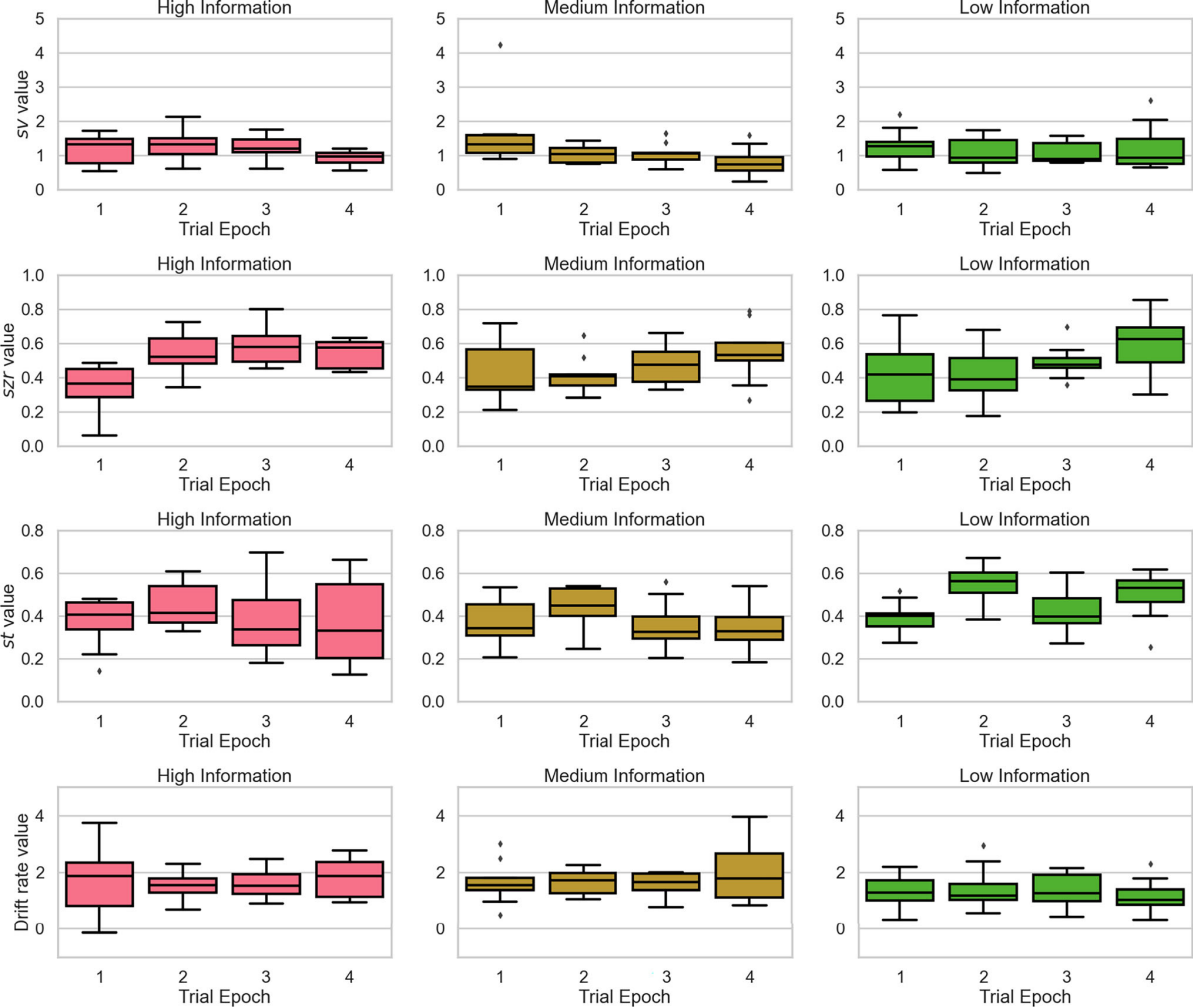
Box plots depicting changes in between trial variability parameters of the *drift variability model* (top panels), *start-point variability model* (second row of panels), *non-decision time variability model* (third row of panels), and the drift and $\alpha$ varying model (bottom panels) for the High (left column of panels), Medium (middle column of panels), and Low Information (right column of panels) conditions across trial epochs

Figure 5 shows how the jump parameter, $\alpha$, varies across trial epochs in each condition. In the ‘low’ and ‘medium’ information conditions, changes in $\alpha$ are not clear-cut across trial epochs. In the ‘high’ information condition, however, there are general increases in $\alpha$ (i.e., a 17% increase in the last epoch with respect to the first epoch) alongside lower levels of variability across trial epochs. Although there is substantial overlap in the distributions of $\alpha$, particularly in the earliest epochs, the overall pattern suggests greater stability in strategy selection and/or execution with increasing practice. Indeed, the pattern of increasing $\alpha$ values in this condition means that the behavior of the model more closely approximates the standard DDM with increasing practice. If $\alpha$ tends to increase with practice, it is perhaps unsurprising why the DDM has proved so successful: in highly practiced participants, whose data are often used to test the standard model, within-trial accumulation noise will be well characterized by a Gaussian process (or equivalently, an $\alpha$-stable distribution, where $\alpha$=2). We note, however, that the estimates of $\alpha$ remain lower than 2, suggesting that decision stability has yet to plateau after a single session of practice on a task. This leaves open the possibility that analysis with the LF model may even be beneficial in more highly practiced individuals.

We note that the changes in $\alpha$ did not come at the expense of the changes in the decision threshold reported by Evans and Brown (2017). Figure 6 shows systematic reductions in decision threshold across trial epochs, replicating their core result.

We next considered the performance of the four control models that allowed either drift rate variability ($s_{v}$), start-point variability ($s_{zr}$), non-decision time variability ($s_{t}$), or both mean drift rate (*v*) and $\alpha$ to differ across epochs. Changes in each model’s namesake parameter across trial epochs are shown in Fig. 7. It is clear from the figure that there are no systematic changes in non-decision time variability across trial epochs (third row of panels). This suggests that there is no tradeoff between $\alpha$ and non-decision time variability. However, there appear to be systematic changes in drift rate variability (top panels) as well as start-point variability (second row of panels) as a function of practice, which raises concerns about whether the changes in $\alpha$ reported for the $\alpha$ varying model are simply mimicking changes in these between-trial variability parameters that are available to the standard DDM.

Turning first to the drift variability model, there is a decreasing trend in drift rate variability, $s_{v}$, across trial epochs in both the ‘medium’ and ‘high’ information conditions. This decreasing pattern is only significant in the ‘medium’ condition. One interpretation of this trend is that it reflects increased consistency in how the stimulus is encoded as people become more practiced with the task. This interpretation aligns well with the interpretation of $\alpha$ proposed by Voss et al. (2019) (i.e., that it reflects consistent strategy use and/or the capacity to reliably execute a given decision strategy). It is possible, then, that the changes in $\alpha$ seen with the $\alpha$ varying model are actually mimicking reductions in drift rate variability. Based on the model selection results reported in Table 2, and the relatively poor performance of the drift variability model, it appears that the degree of mimicry between $\alpha$ and $s_{v}$ is only partial. Given that changes in $s_{v}$ are insufficient to explain the effects of gradual increases in $\alpha$ with practice, we tentatively conclude that changes in $\alpha$ may reflect more stable reliance on a well-learned decision strategy, consistent with the claims of Voss et al. (2019), and that these changes are not restricted to consistency in strategy selection or application across trials.

Turning to the start-point variability model, there is a clear increasing trend in start-point variability, $s_{zr}$ across trial epochs in all ‘low’, ‘medium’, and ‘high’ information conditions. The overall pattern of change, especially in the high information condition, is similar to the changes observed in $\alpha$ for the $\alpha$ varying model. The effect of increasing start-point variability, however, runs counter to the effect of increasing $\alpha$ in the LF model, and so we think it is unlikely that the changes in these model parameters observed here reflect any meaningful tradeoff. Specifically, start-point variability is important for allowing the standard DDM to fit patterns of data where error responses are, on average, faster than correct responses (Ratcliff & Rouder, 1998). While fits to this fast-error pattern have been used to support the LF model (Voss et al., 2019), these fits require *lower* values of the $\alpha$ parameter. The increases in $\alpha$ shown by the $\alpha$ varying model imply a progressively lower rate of fast errors with practice, whereas the increasing pattern of $s_{zr}$ shown by the start-point variability model implies an increasing rate of fast errors with practice. On balance, we conclude that changes in start-point variability do not provide a viable explanation of changes in the $\alpha$ parameter.

Finally, for the drift and $\alpha$ varying model, we see the same pattern of changes in the $\alpha$ parameter across trial epochs in the high information condition (see lower panels of Figure 5). However, there were no systematic changes in mean drift rate for any of the information conditions (see bottom panels of Fig. 7). Taken together, this suggests that the effects of $\alpha$ are not duplicating effects better attributed to variation in drift rate. Of note, for these data, (Evans & Brown, 2017) also found little support for changes in drift rate over epoch. Overall, we conclude that the $\alpha$ varying model was the best performing model we considered. Model selection based on both AIC and BIC further support this (Table 3). Parameter estimates for each model are shown in Table 3.

## Discussion

Our primary aim with the current study was to investigate the assumptions made by the LF model regarding within-trial evidence accumulation dynamics. Specifically, we provided a critical investigation of the psychological plausibility of the sudden large jumps in evidence accumulation that the model allows via its $\alpha$ parameter. While previous work has shown that the LF model provides a good fit to data exhibiting patterns of fast errors (Voss et al., 2019), it is not clear whether this is simply due to the increased flexibility afforded by $\alpha$, or if the LF model provides a more accurate characterization of the underlying psychological process. Voss et al. (2019) proposed that $\alpha$ might be interpreted as reflecting the stability or consistency with which an individual selects or executes a given decision strategy (e.g., jumps in evidence accumulation, due to $\alpha$, may reflect sudden shifts in the source of evidence when people are less adept at parsing the stimulus). This conjecture was the subject of our investigation.

We reasoned that if $\alpha$ could be interpreted psychologically along the lines proposed by Voss et al. (2019), we should be able to observe systematic changes in this parameter, as people refine a decision strategy and become more experienced in executing it. That is, we might expect systematic increases in the $\alpha$ parameter as a function of practice. We reanalyzed practice data from a study by Evans and Brown (2017), who showed different patterns of improvement as a function of how informative the performance feedback they received was. Our analysis of these data with the LF model showed that when $\alpha$ was free to vary as a function of practice, as in the $\alpha$ *varying model*, we saw systematic increases in $\alpha$, as a function of practice. This increasing pattern was most evident in the high information condition, where participants received the most guidance on how to refine their decision strategy over time. Our result coheres well with the conjecture of Voss et al. (2019), as the high information condition would be where one would expect participants to have refined their decision strategy to the greatest degree.

**Table 3:** Estimated parameters of the models for each condition. Mean parameter estimates across participants are reported along with the variance in estimates in parentheses

Model	Condition	$a_{1}$	$a_{2}$	$a_{3}$	$a_{4}$	*v*	$z_{r}$	$t_{0}$	$\alpha _{1}$	$\alpha _{2}$	$\alpha _{3}$	$\alpha _{4}$			
Model 1	High Info.	4.51 (6.72)	1.79 (0.09)	1.91 (0.26)	1.43 (0.15)	1.30 (0.37)	0.50 (0.01)	0.46 (0.01)	1.33 (0.05)	1.39 (0.03)	1.47 (0.02)	1.50 (0.01)	–		
	Medium Info.	3.00 (2.56)	2.09 (0.59)	1.91 (0.45)	1.62 (0.20)	1.71 (0.35)	0.52 (0.02)	0.47 (0.006)	1.29 (0.02)	1.46 (0.02)	1.46 (0.04)	1.48 (0.05)	–		
	Low Info.	2.81 (2.69)	2.46 (0.56)	2.10 (0.42)	1.78 (0.49)	1.12 (0.18)	0.51 (0.01)	0.49 (0.004)	1.42 (0.02)	1.49 (0.04)	1.55 (0.006)	1.44 (0.01)	–		
		$a_{1}$	$a_{2}$	$a_{3}$	$a_{4}$	*v*	$z_{r}$	$t_{0}$	$\alpha$	$s_{v1}$	$s_{v2}$	$s_{v3}$	$s_{v4}$		
Model 2	High Info.	5.81 (16.36)	1.76 (0.16)	1.88 (0.50)	1.68 (0.35)	2.31 (1.11)	0.37 (0.01)	0.41 (0.01)	1.29 (0.01)	1.16 (0.20)	1.34 (0.21)	1.25 (0.11)	0.92 (0.04)		
	Medium Info.	3.18 (3.08)	2.12 (0.42)	2.06 (0.46)	2.01 (0.21)	2.61 (0.63)	0.40 (0.009)	0.44 (0.008)	1.28 (0.01)	1.57 (1.04)	1.03 (0.05)	1.04 (0.09)	0.82 (0.18)		
	Low Info.	3.29 (4.74)	2.46 (0.54)	2.34 (0.60)	2.06 (0.54)	1.89 (0.39)	0.39 (0.004)	0.46 (0.003)	1.32 (0.02)	1.23 (0.22)	1.05 (0.20)	1.06 (0.09)	1.21 (0.41)		
		$a_{1}$	$a_{2}$	$a_{3}$	$a_{4}$	*v*	$z_{r}$	$t_{0}$	$\alpha$	$s_{zr1}$	$s_{zr2}$	$s_{zr3}$	$s_{zr4}$		
Model 3	High Info.	4.96 (6.06)	1.80 (0.17)	1.75 (0.43)	1.69 (0.21)	1.47 (0.78)	0.59 (0.01)	0.53 (0.01)	1.17 (0.01)	0.33 (0.02)	0.54 (0.01)	0.58 (0.01)	0.54 (0.006)		
	Medium Info.	3.02 (1.96)	2.09 (0.22)	1.78 (0.50)	1.86 (0.20)	1.76 (0.70)	0.59 (0.02)	0.56 (0.005)	1.13 (0.01)	0.41 (0.02)	0.41 (0.01)	0.46 (0.01)	0.54 (0.02)		
	Low Info.	3.04 (3.69)	2.43 (0.23)	2.11 (0.33)	2.05 (0.56)	1.14 (0.34)	0.58 (0.01)	0.60 (0.01)	1.19 (0.01)	0.42 (0.03)	0.41 (0.02)	0.48 (0.007)	0.59 (0.02)		
		$a_{1}$	$a_{2}$	$a_{3}$	$a_{4}$	*v*	$z_{r}$	$t_{0}$	$\alpha$	$s_{t1}$	$s_{t2}$	$s_{t3}$	$s_{t4}$		
Model 4	High Info.	4.90 (50.26)	1.33 (0.25)	1.46 (0.27)	1.28 (0.34)	1.31 (0.62)	0.64 (0.02)	0.62 (0.01)	1.13 (0.01)	0.37 (0.01)	0.44 (0.01)	0.37 (0.02)	0.37 (0.04)		
	Medium Info.	3.20 (3.39)	1.96 (0.89)	1.66 (0.43)	1.71 (0.42)	1.75 (1.05)	0.67 (0.02)	0.63 (0.02)	1.14 (0.01)	0.36 (0.01)	0.43 (0.01)	0.35 (0.01)	0.34 (0.01)		
	Low Info.	2.79 (4.89)	2.03 (0.76)	1.89 (1.11)	1.62 (0.77)	0.99 (0.23)	0.57 (0.02)	0.62 (0.006)	1.19 (0.02)	0.39 (0.004)	0.54 (0.007)	0.41 (0.009)	0.50 (0.01)		
		$a_{1}$	$a_{2}$	$a_{3}$	$a_{4}$	$z_{r}$	$t_{0}$	$\alpha _{1}$	$\alpha _{2}$	$\alpha _{3}$	$\alpha _{4}$	$v_{1}$	$v_{2}$	$v_{3}$	$v_{4}$
Model 5	High Info.	4.53 (1.42)	1.71 (0.63)	1.90 (0.44)	1.94 (0.25)	0.51 (0.01)	0.45 (0.007)	1.42 (0.02)	1.44 (0.01)	1.47 (0.01)	1.54 (0.003)	1.71 (1.42)	1.50 (0.22)	1.61 (0.28)	1.81 (0.48)
	Medium Info.	2.95 (1.74)	1.96 (0.42)	1.81 (0.26)	2.31 (0.50)	0.56 (0.01)	0.49 (0.01)	1.47 (0.03)	1.57 (0.01)	1.49 (0.01)	1.47 (0.02)	1.67 (0.56)	1.66 (0.16)	1.61 (0.16)	2.01 (1.07)
	Low Info.	3.09 (1.78)	2.50 (0.54)	2.31 (0.25)	2.09 (0.70)	0.52 (0.01)	0.53 (0.01)	1.47 (0.03)	1.45 (0.01)	1.50 (0.01)	1.49 (0.02)	1.34 (0.29)	1.40 (0.48)	1.38 (0.31)	1.16 (0.30)

*Note*. Model 1 = $\alpha$ Varying Model; Model 2 = Drift Variability Model; Model 3 = Start-Point Variability Model; Model 4 = Non-Decision Time Variability Model; Model 5 = Drift & $\alpha$ Varying Model

We also considered whether the changes in $\alpha$ we observed could simply reflect a tradeoff with some other model parameter that is available to the standard DDM (viz. mean drift rate, or between-trial variability in either drift rate, starting point, or non-decision time). Our analysis showed that while there is some overlap between changes in $\alpha$ and changes in trial-to-trial variability in drift rate, $s_{v}$, the changes in the drift rate variability parameter alone are insufficient to explain the benefits of allowing $\alpha$ to vary with practice. Notably, we found no systematic changes in the mean drift rate across trial epochs. The stability of drift rates was observed alongside changes in $\alpha$ in the high information condition, suggesting that $\alpha$ does not simply trade-off with other diffusion model parameters when fit to choice data.

It is worth noting some limitations with our study, such as computational limitations (i.e., having to aggregate trial blocks into epochs to avoid problems with parameter recovery) and more importantly, a relatively low number of participants in the condition demonstrating variation in $\alpha$ as a function of practice. Specifically, there were only 11 participants in the high-information condition, necessitating caution in the interpretation of our result. Replicating these results with a larger sample will be important to establish the robustness of interpreting $\alpha$ as a potential marker of decision heterogeneity within an individual. Another important point of caution relates to the variability in epoch-on-epoch changes in the $\alpha$ parameter. In both models that allowed $\alpha$ to vary across epochs (i.e., Model 1 and Model 5), there was considerable overlap in the distributions across epochs, particularly in the earlier parts of the task. The increases we observed were mostly restricted to the the final trial epoch. The relatively noisy changes in $\alpha$, including non-monotonic changes across the first two epochs, necessitate some caution in interpretation, though we do note that such changes are consistent with the notion of a greater variety of decision strategies/hypotheses an observer might pursue in the earlier parts of the task.

### Lévy flights or Wiener diffusion?

Our analysis provides further preliminary support for using LFs as a model of human decision-making. Given the similarities between the LF model and the standard Wiener diffusion model (Ratcliff, 1978; Ratcliff & McKoon, 2008; Ratcliff & Rouder, 1998), it is reasonable to ask whether the LF model can tell us anything more about decision-making than what can be achieved through a standard diffusion model analysis. The answer to this question depends heavily on the extent to which accumulation noise in the decision process deviates from standard Gaussian assumptions. This will likely depend on the level of analysis one adopts when modeling the decision process. For example, at the lower level of spiking neurons, the noise will likely be more heavy-tailed than assumed by a Gaussian (e.g., Poisson noise provides a good characterization at this level; Wang, 2002). However, the aggregate properties of low-level models with non-Gaussian noise can be shown to be approximated well by diffusion models that assume Gaussian noise at evidence accumulation (Smith, 2010). It follows that at the level of overt behavior, standard diffusion models may be sufficient. A potential caveat though concerns situations where responses are collected from relatively novice observers or those who are inexperienced with a task. Here, the conjecture of Voss et al. (2019), that heavy-tailed accumulation noise might provide a better account may provide additional insight. If novice or inexperienced observers with a task are more variable in the response strategies they adopt, a LF analysis should show increased variability in accumulation noise via estimates of $\alpha < 2$. Indeed, our reanalysis of the Evans and Brown (2017) data support this. We suspect that as observers become more experienced with a task—or become more single-minded in the execution of a preferred response strategy—their performance will be better approximated by a standard Wiener diffusion model. With less experienced observers, a LF model may provide a good way of indirectly assessing variability in response strategy; something that typically requires analysis of verbal reports collected after-the-fact from participants. This sort of analysis opens the door to then developing more explicit theories of the variety of decision strategies people may rely on when attempting a new task (e.g., by assuming a mixture of differently configured Wiener processes). It may also provide a principled first step towards developing mechanistic models of how one chooses among these different candidate strategies on a trial-by-trial basis (e.g., Rieskamp & Otto, 2006).

If it is the case that the LF model has the potential to provide insights that are not as directly attained through standard diffusion model analysis, another question is whether LF analyses cast doubt on conclusions based on existing diffusion model analyses. That is, is there reason to mistrust processing insights gained by existing diffusion model analyses? To this, we think the answer is firmly no. Like other existing sequential sampling models, the LF model decomposes behavioral performance into latent variables corresponding to quality of evidence (drift rate), caution (boundary separation), and non-decision time. Donkin et al. (2011) found that application of two quite different model architectures—Ratcliff’s diffusion model (Ratcliff & McKoon, 2008) and the linear ballistic accumulator (Brown & Heathcote, 2008)—would lead to similar conclusions regarding latent variables responsible for generating effects in data. The similarity of the LF model to the standard DDM leads us to believe that it is highly likely that inferences about whether an effect is driven by (say) quality of evidence versus (say) response caution will not depend strongly on which model is applied to data.

It is also important to consider the implications of the $\alpha$ parameter as an indicator of a mixture of decision strategies. Unless reliance on different strategies varies on a moment-to-moment basis, or alternatively, if the outputs of all strategies simultaneously feed into the decision stage resulting in superimposed evidence (e.g., Lee & Sewell, 2023; Ulrich et al., 2015), the LF model might mischaracterize the properties of the component decision strategies. For example, the singular drift rate summarized by the LF model may not reflect the distribution of drift rates of different (say) Wiener diffusion processes associated with different strategies. Since $\alpha$ affects *within-trial* evidence accumulation dynamics as opposed to *between-trial variability in strategy selection*, the mixture of strategies is necessarily modeled as if it were a single aggregate strategy. We think it is reasonable to view selection of a decision strategy primarily as a between-trial phenomenon, rather than something that can vary on a moment-to-moment timescale, and so a LF analysis will still require further investigation into how different decision strategies ought to be characterized and selected and/or combined to fit a given data set. This issue similarly besets any DDM analysis of a data set where responses reflect a mixture of strategies. On balance, we view the potential insights that can be gained by a LF analysis to complement insights already obtained through existing DDM analyses. In both cases, the results provide guidance on further theoretical work that needs to occur to provide a fuller characterization of psychological processing. For example, both DDM and LF analyses can shed light on how "theories of drift rates" need to be articulated. The LF model can potentially go further in demonstrating a need to develop a set of theories of drift rates to characterize different competing decision strategies that novices on a task might be selecting from.

### Lévy flights models: current status and future directions.

How then should we view the LF model, and the assumptions it makes regarding the nature of evidence accumulation? On the one hand, our application of the model to the practice data of Evans and Brown (2017) suggests that systematic changes in $\alpha$ unfold in a manner that is sensible and theoretically consistent with our understanding of how task performance improves as a function of practice. It is therefore tempting to conclude that the jumps in evidence accumulation implied by the LF model are indeed real, and that the $\alpha$ parameter indexes something akin to the “stability" of a decision strategy. We believe such a conclusion would be premature, as, on the other hand, there is a broader question of whether the stability in applying, selecting, or executing a decision strategy over the course of an experiment is best represented as a *within-trial* noise parameter controlling moment-to-moment perturbations in evidence accumulation, or if it could be better characterized in some other way. If refinements of decision strategy serve to limit the noise that enters the decision process—perhaps by allowing observers to focus attention on the most relevant or diagnostic parts of a stimulus, or by rendering the observer less vulnerable to momentary distraction—then it is perhaps appropriate to interpret $\alpha$ as an index of decision stability. If, however, the evolution of decision strategies is more consistent with a process of selecting and testing candidate decision hypotheses, it would be more sensible to consider decision stability as a phenomenon that is best understood as occurring *across trials*, rather than varying over the course of a single decision within a trial. If decision instability is more akin to discrete selection of candidate response strategies across trials, then the early stages of practice—where estimates of $\alpha$ are low—may better be characterized by a probability mixture of Wiener diffusion processes, characterized by drift rate distributions with different means and standard deviations. These more detailed comparisons await future research. A logical next step from the current research would be to examine other data sets that have been used to assess learning- or experience-related changes in diffusion model parameters over time (e.g., Dutilh et al., 2009; Lerche & Voss, 2017) to see if similar changes in $\alpha$ arise there as well. One particularly fruitful direction for future research is to better characterize the relationship between the LF $\alpha$ parameter and existing parameters in the DDM. Our comparison of different model variations above suggest that the effects of the $\alpha$ parameter are not duplicating the effects of other between-trial variability parameters that are commonly included in the DDM (viz. trial-to-trial variability in drift rates, starting point, and non-decision time). A cross-fitting exercise similar to the one conducted by Donkin et al. (2011) would clarify the relationship between $\alpha$ and other more established model parameters while also providing suggestions about the kinds of signatures in data that $\alpha$ might especially be sensitive to (e.g., patterns of fast errors, as described by Voss et al. 2019).

Given the open question of whether the heavy-tailed accumulation noise described by $\alpha$ best reflects between- or within-trial processing dynamics, there is an urgent need to understand moment-to-moment volatility of people’s decision strategies and/or the extent to which people can maintain focus on a single source of evidence while making a decision. Attentional selection paradigms might present an ideal setting for exploring this question, as both relevant and irrelevant information is presented, requiring the observer to filter out the irrelevant information in order to respond correctly. Andersen et al. (2009) used a dot motion task where dots of two different colors were spatially overlaid with one another. In order to report the correct direction of motion, observers needed to selectively ignore the irrelevant colored dots. If $\alpha$ is sensitive to people’s capacity to focus attention on a single source of perceptual evidence, individual differences in this parameter should correlate with other assays of attentional control.

Another future direction for research will be to examine how multiple sources of information might influence decision-making in non-perceptual domains. Wang et al. (2022) have suggested that jumps in evidence accumulation may reflect parallel activation of multiple information sources from episodic memory. This idea can be investigated in more detail, for example, in learning tasks where information in memory about multiple stimuli must be combined to form a decision. In these contexts, the LF model might be used to identify within-trial dynamics in the influence of multiple memory-based sources of evidence on decision-making (e.g., information specific to the stimulus vs. information about the task structure as a whole). To the extent that different parallel streams of information are being accumulated will highlight the utility of using the LF model as a tool for assessing task-related processing.

### Conclusion

In this article, we investigated the conjecture of Voss et al. (2019) that the LF $\alpha$ parameter might provide a measure of decision stability. We observed systematic changes in $\alpha$ as a function of practice in a data set previously published by Evans and Brown (2017). While the question of exactly what $\alpha$ corresponds to psychologically remains open, for now, we think it is prudent to conclude that the LF $\alpha$ parameter reflects a psychologically meaningful construct. However, future research will need to carefully investigate whether the construct is best represented in formal models of decision-making as a between- or within-trial phenomenon. Our analysis of practice data using the LF model suggests that more diagnostic tests of the model may require participants who are less practiced on tasks, as individuals in the early stages of practice will be most likely to be trying to discern the best way to perform and will be the most open to trying a number of different approaches.

## Supplementary Information

Below is the link to the electronic supplementary material.

Supplementary file 1 (pdf 870 KB)

## Data Availability

All data and codes are available on: https://osf.io/fgupj
