# Supplementary material for: Are there jumps in evidence accumulation, and what, if anything, do they reflect psychologically? An analysis of Lévy Flights models of decision-making
Source: Psychon Bull Rev. 2023 Jul 19;31(1):32–48. doi: 10.3758/s13423-023-02284-4 (PMC11420318; doi:10.3758/s13423-023-02284-4)
Supplement: Supplementary file 1 — (pdf 870 KB) [file 13423_2023_2284_MOESM1_ESM.pdf]

Supplementary Materials

Here, we present model predictions and parameter estimates for the drift variability, start-point variability, non-decision time variability, and drift and α varying models.

Model predictions for the drift variability, start-point variability, non-decision time variability, and drift and α varying models

Model prediction for the four control models described in the main text are shown below. Figures A.1, A.2, A.3, and A.4 show model predictions for the *drift variability*, *start-point variability*, *non-decision time variability*, and drift and α varying models respectively.

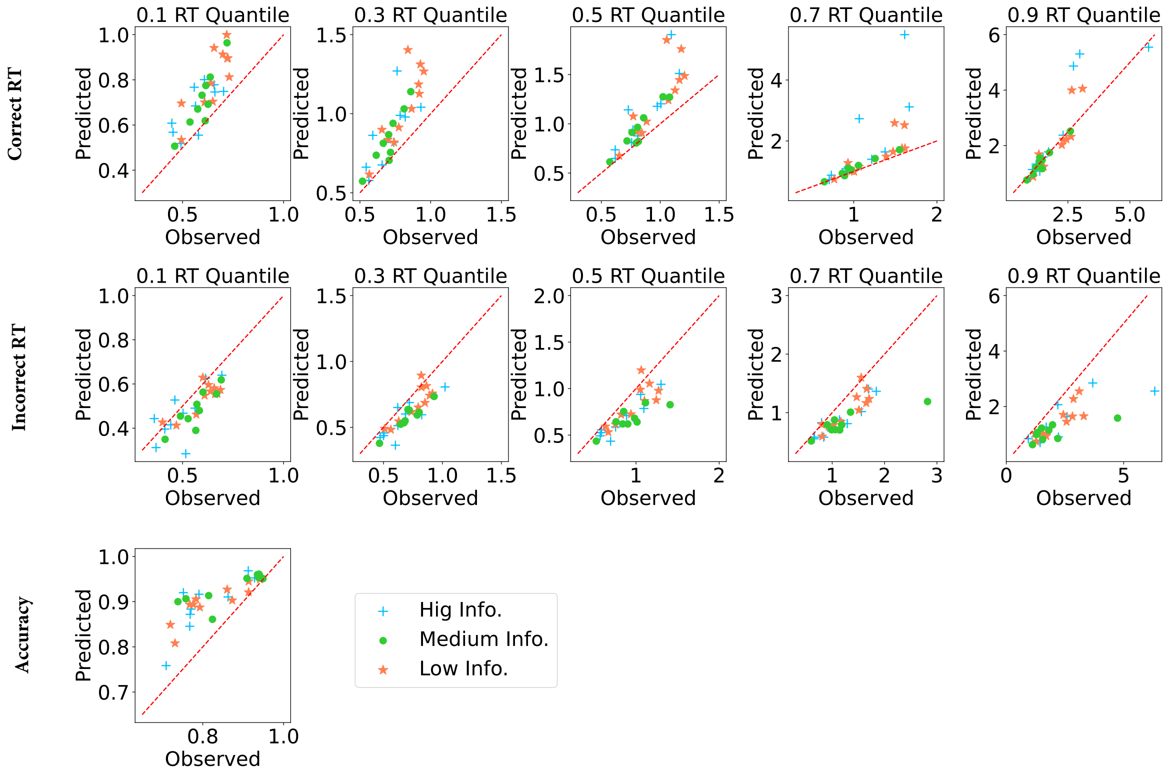

Figure A.1. Predictions of the *drift variability model*. The top panel shows the 0.1, 0.3, 0.5, 0.7, and 0.9 correct response time quantiles. The middle panel shows the corresponding quantiles for incorrect responses. The bottom panel shows accuracy predictions. Each point shows one participant.

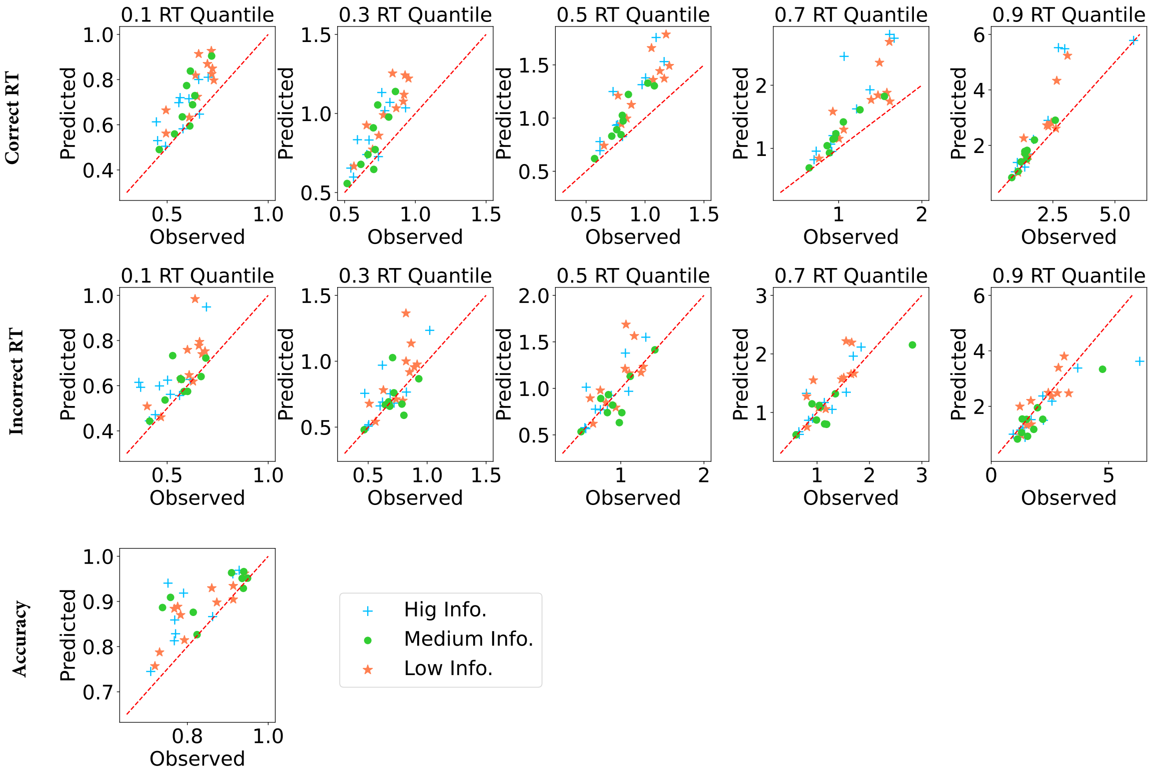

Figure A.2. Predictions of the *start-point variability model.* The top panel shows the 0.1, 0.3, 0.5, 0.7, and 0.9 correct response time quantiles. The middle panel shows the corresponding quantiles for incorrect responses. The bottom panel shows accuracy predictions Each point shows one participant.

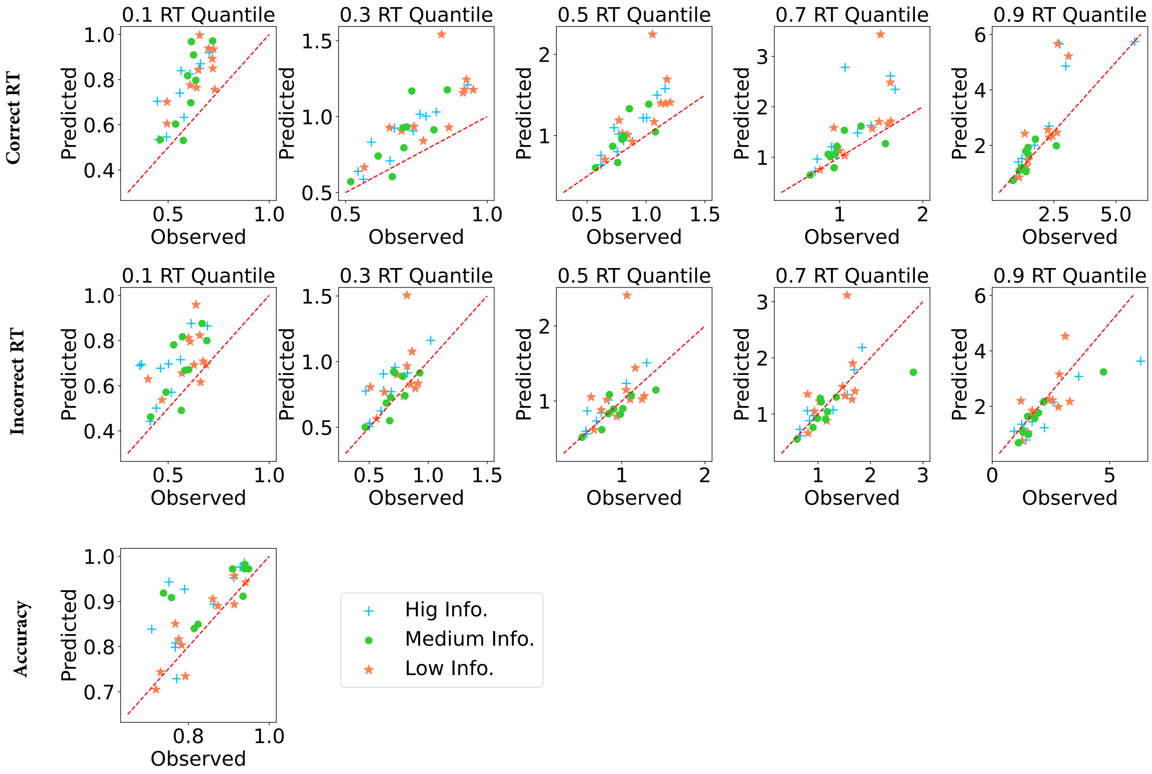

Figure A.3. Predictions of the *non-decision time variability model*. The top panel shows the 0.1, 0.3, 0.5, 0.7, and 0.9 correct response time quantiles. The middle panel shows the corresponding quantiles for incorrect responses. The bottom panel shows accuracy predictions Each point shows one participant.

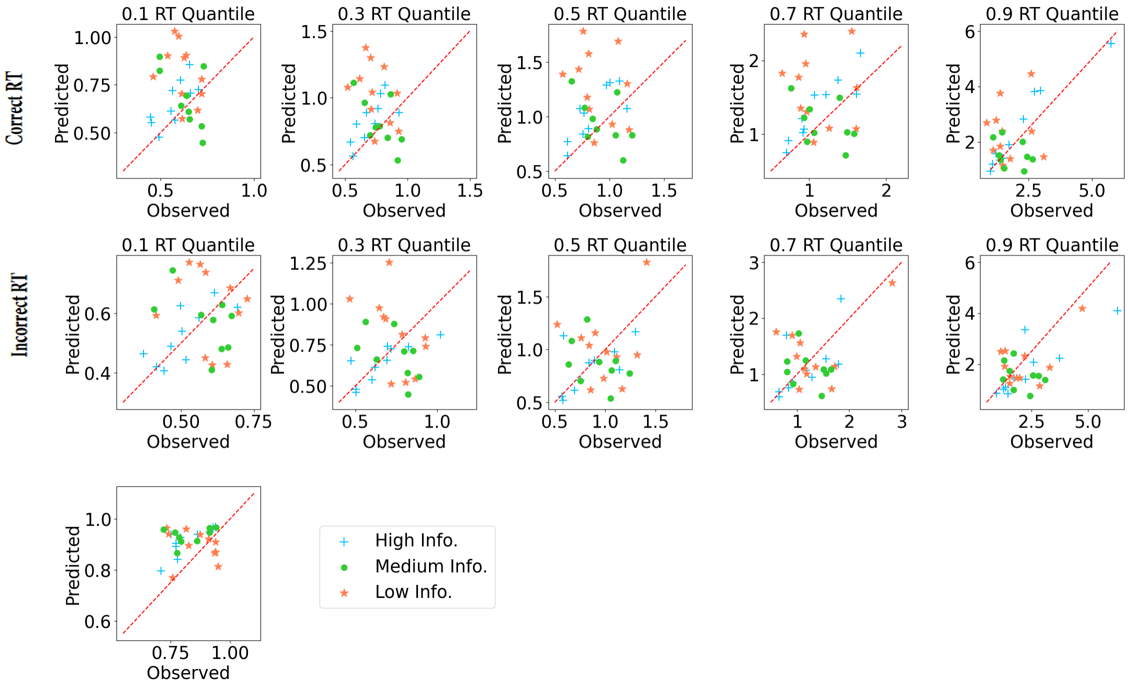

Figure A.4. Predictions of the *Drift & α varying model*. The top panel shows the 0.1, 0.3, 0.5, 0.7, and 0.9 correct response time quantiles. The middle panel shows the corresponding quantiles for incorrect responses. The bottom panel shows accuracy predictions Each point shows one participant.

Estimated decision threshold parameter of drift variability, start-point variability, and non-decision time variability models

Figure A.5 shows estimates of the decision threshold parameter (a) across epochs for the drift variability, start-point variability, and non-decision time variability models models.

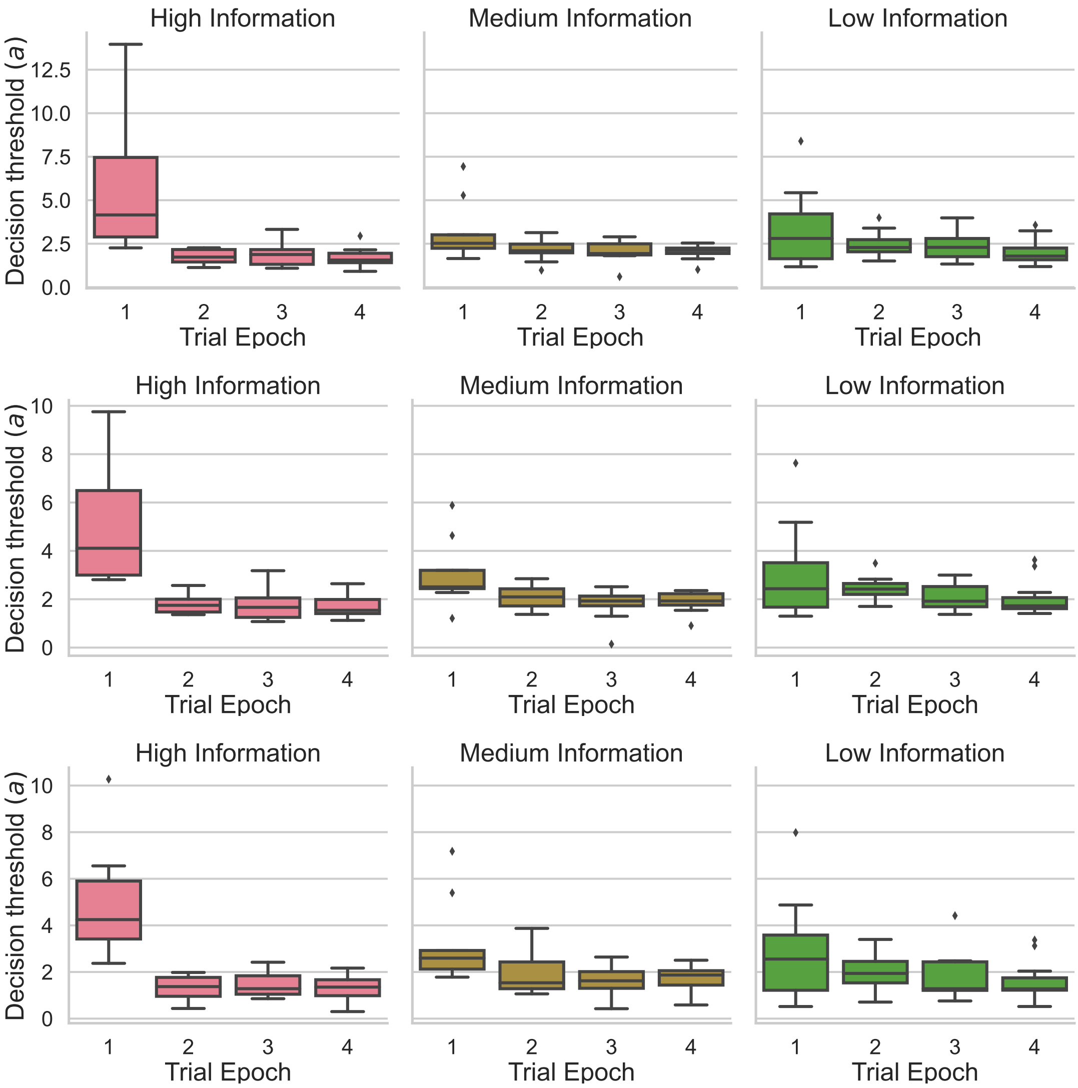

Figure A.5. Box plots depicting changes in the decision threshold parameter (a), across trial epochs for the drift variability model (top panel), start-point variability model (middle panel), and non-decision time variability model (button panel).

Estimated parameter from individual participant fits

We now present estimated parameters from fitting all five models to individual participant data across trial epochs in Figures A.6 to A.17. Each model for each information condition is depicted in a separate figure.

α Varying Model

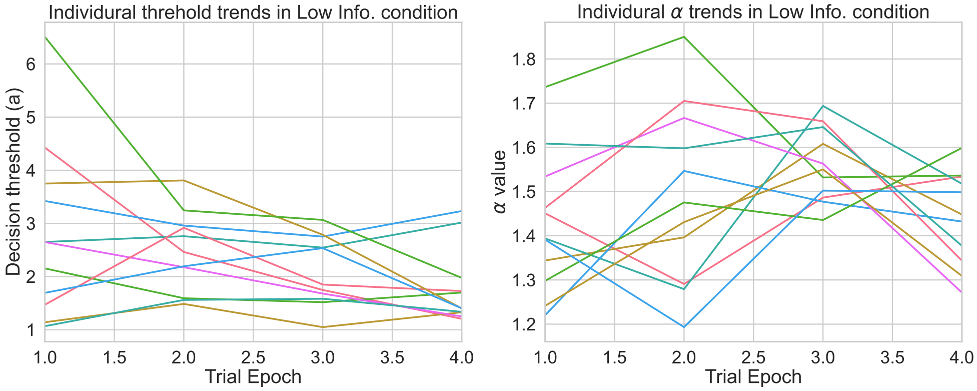

Figure A.6. Individual parameter estimates of the α *varying model*, for the participants in 'Low Information' condition for threshold (left panel) and α (right panel). Each line shows one participant. We refer the reader onto the online version of this article for the color version of this figure.

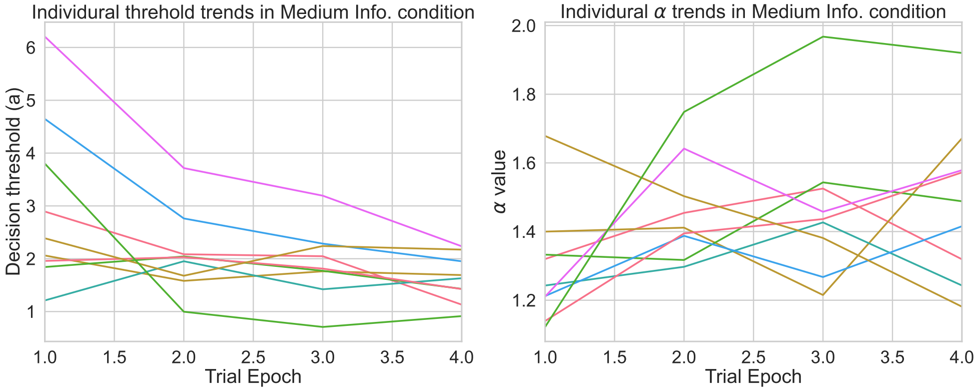

Figure A.7. Individual parameter estimates of the α *varying model*, for the participants in 'Medium Information' condition for threshold (left panel) and α (right panel). Each line shows one participant. We refer the reader onto the online version of this article for the color version of this figure.

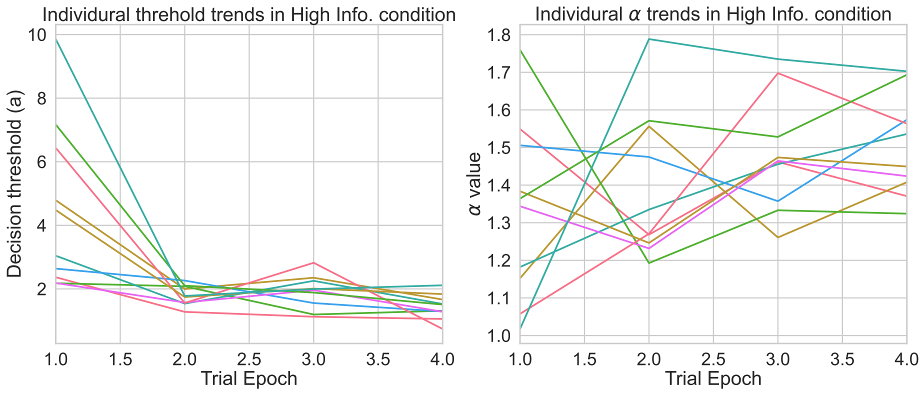

Figure A.8. Individual parameter estimates of the α *varying model*, for the participants in 'High Information' condition for threshold (left panel) and α (right panel). Each line shows one participant. We refer the reader onto the online version of this article for the color version of this figure.

Drift Variability Model

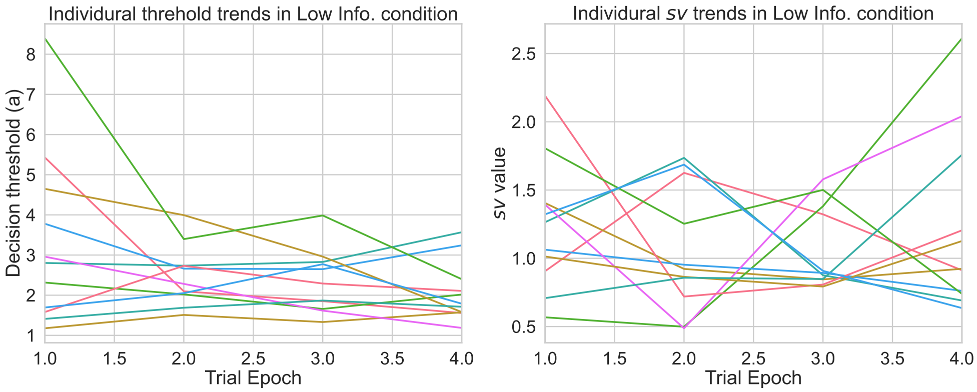

Figure A.9. Individual parameter estimates of the *drift variability model*, for the participants in 'Low Information' condition for threshold (left panel) and sv (right panel). Each line shows one participant. We refer the reader onto the online version of this article for the color version of this figure.

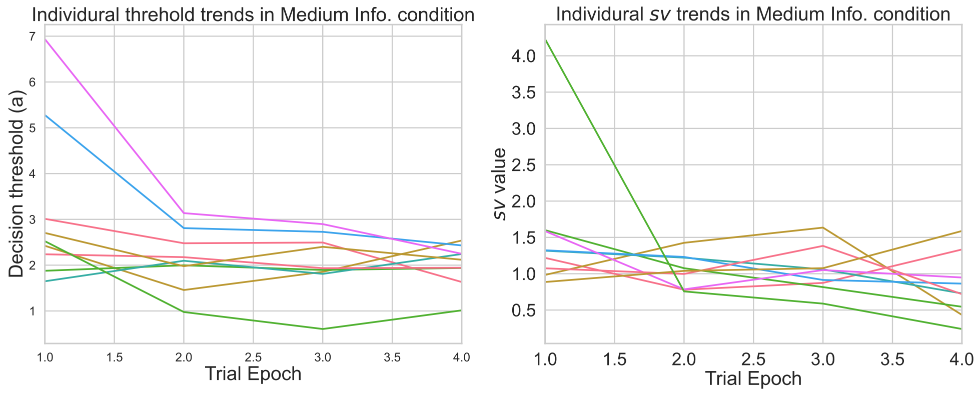

Figure A.10. Individual parameter estimates of the *drift variability model*, for the participants in 'Medium Information' condition for threshold (left panel) and *sv* (right panel). Each line shows one participant. We refer the reader onto the online version of this article for the color version of this figure.

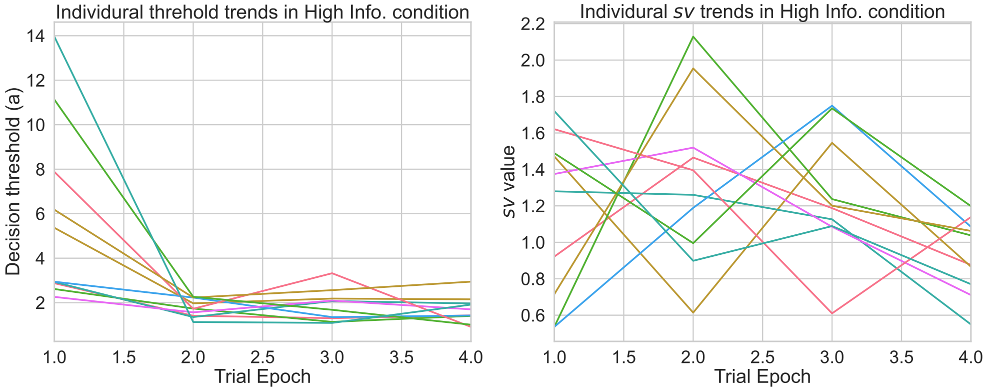

Figure A.11. Individual parameter estimates of the *drift variability model*, for the participants in 'High Information' condition for threshold (left panel) and *sv* (right panel). Each line shows one participant. We refer the reader onto the online version of this article for the color version of this figure.

Start-Point Variability Model

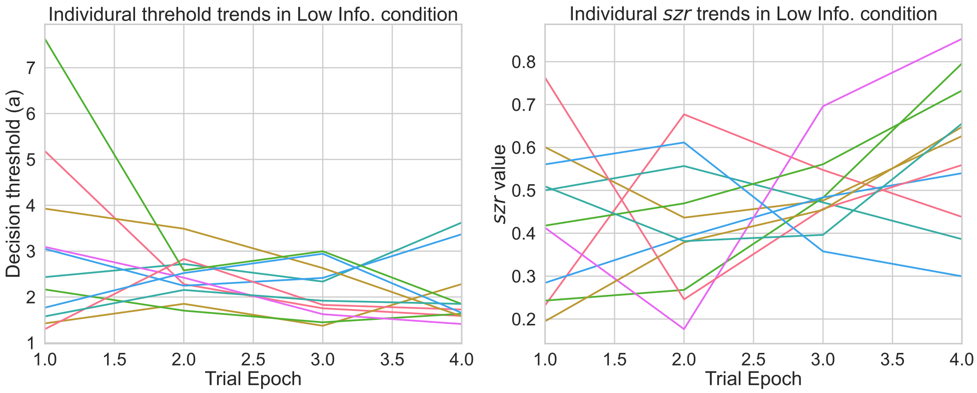

Figure A.12. Individual parameter estimates of the *start-point variability model*, for the participants in 'Low Information' condition for threshold (left panel) and *szr* (right panel). Each line shows one participant. We refer the reader onto the online version of this article for the color version of this figure.

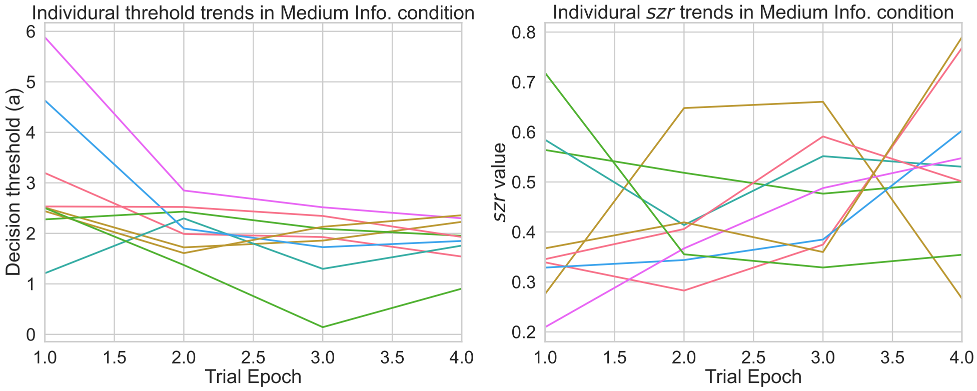

Figure A.13. Individual parameter estimates of the *start-point variability model*, for the participants in 'Medium Information' condition for threshold (left panel) and *szr* (right panel). Each line shows one participant. We refer the reader onto the online version of this article for the color version of this figure.

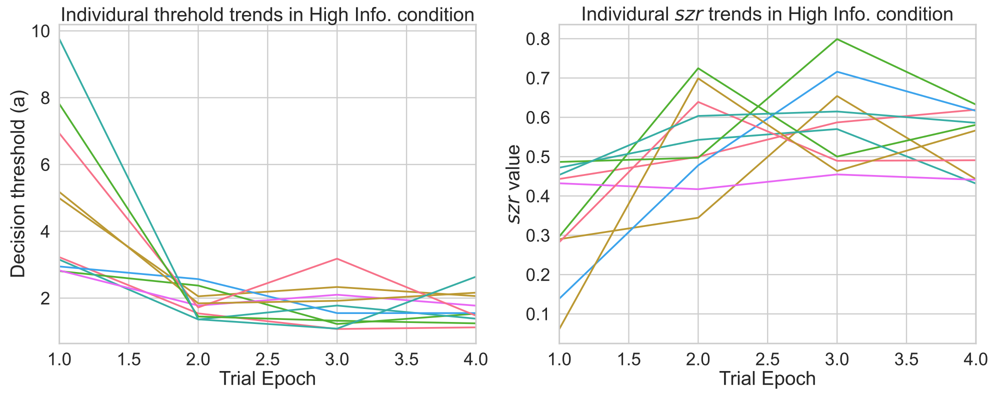

Figure A.14. Individual parameter estimates of the *start-point variability model*, for the participants in 'High Information' condition for threshold (left panel) and *szr* (right panel). Each line shows one participant. We refer the reader onto the online version of this article for the color version of this figure.

Non-Decision Time Variability Model

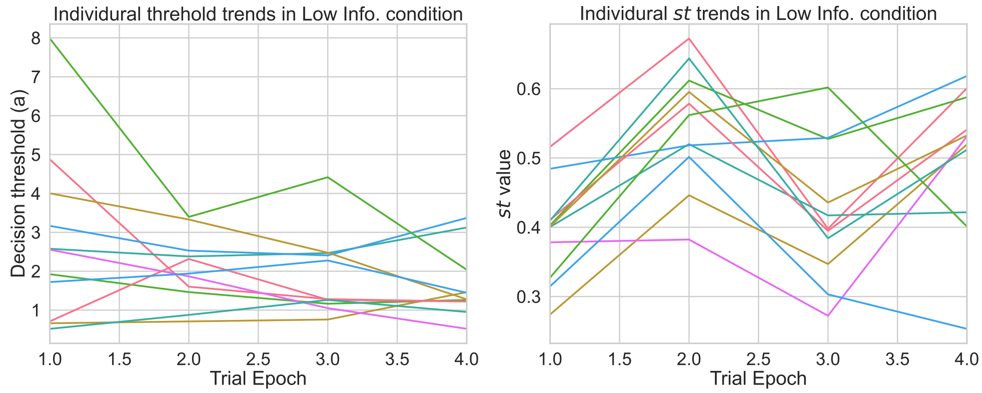

Figure A.15. Individual parameter estimates of the *non-decision time variability model*, for the participants in 'Low Information' condition for threshold (left panel) and *st* (right panel). Each line shows one participant. We refer the reader onto the online version of this article for the color version of this figure.

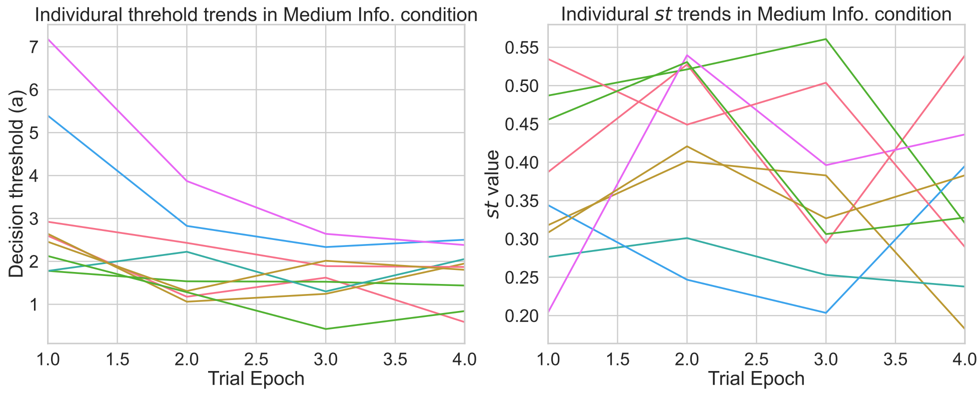

Figure A.16. The recovered parameters of the *non-decision time variability model*, for the participants in 'Medium Information' condition for threshold (left panel) and *st* (right panel). Each line shows one participant. We refer the reader onto the online version of this article for the color version of this figure.

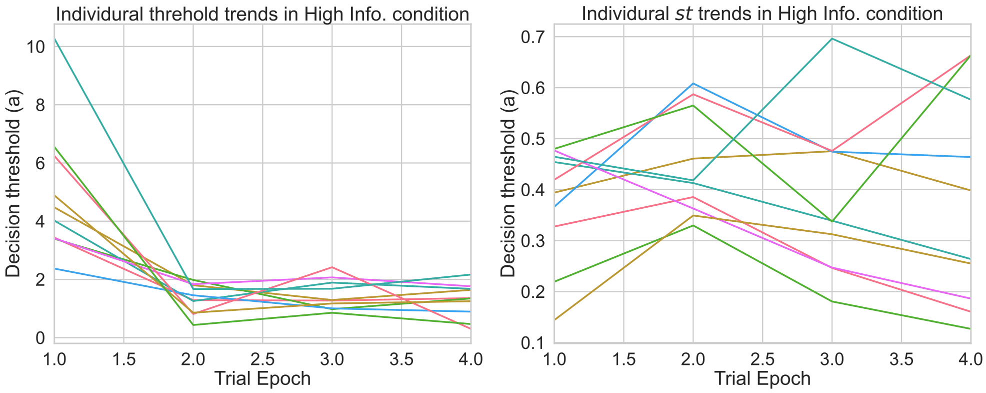

Figure A.17. The recovered parameters of the *non-decision time variability model*, for the participants in 'High Information' condition for threshold (left panel) and *st* (right panel). Each line shows one participant. We refer the reader onto the online version of this article for the color version of this figure.

Drift & α Varying Model

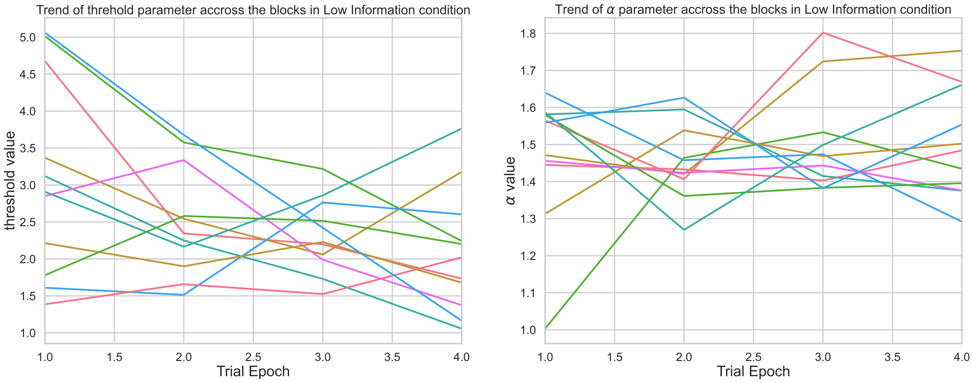

Figure A.18. Individual parameter estimates of the *drift & α varying model*, for the participants in 'Low Information' condition for threshold (left panel) and α (right panel). Each line shows one participant. We refer the reader onto the online version of this article for the color version of this figure.

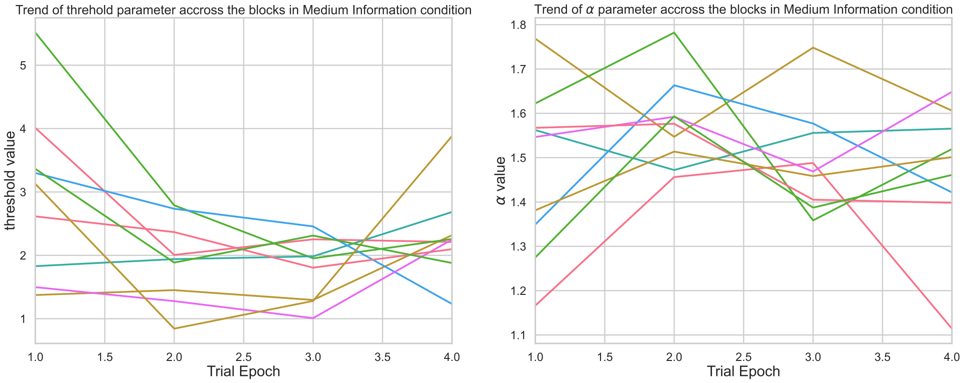

Figure A.19. The recovered parameters of the *drift & α varying model*, for the participants in 'Medium Information' condition for threshold (left panel) and α (right panel). Each line shows one participant. We refer the reader onto the online version of this article for the color version of this figure.

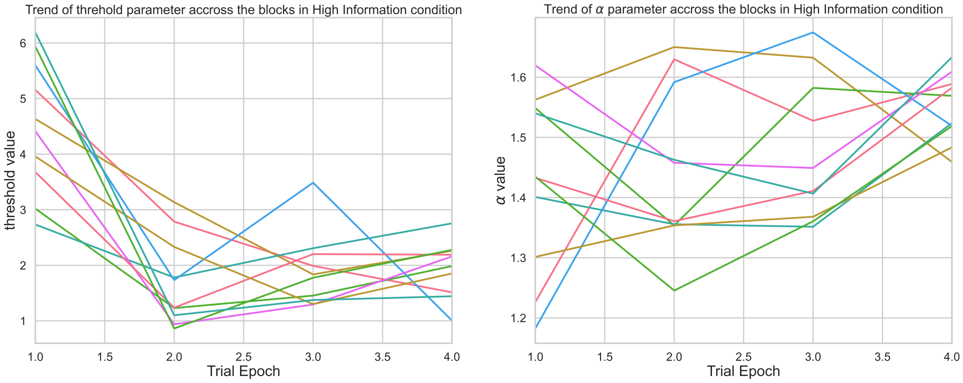

Figure A.20. The recovered parameters of the *drift & α varying model*, for the participants in 'High Information' condition for threshold (left panel) and α (right panel). Each line shows one participant. We refer the reader onto the online version of this article for the color version of this figure.

Simulation Study

To further investigate potential psychological interpretations of the α parameter in the LF model, we examine the behavior of α in different parts of the parameter space of the LF model through simulation. Here, we examine relationships between α and summary measures of simulated data (i.e., accuracy and RT) as a function of different combinations of stimulus and decision factors (i.e., combinations of drift rate and threshold). For these simulations, parameter values were sampled from the following priors:

$$v \sim \mathcal{N}(2.5, 1),$$
$$zr \sim \mathcal{U}(0.3, 0.7),$$
$$a \sim \mathcal{U}(0.5, 3),$$

where $\sim$ means "is distributed as". The non-decision time was fixed for all simulations at $t_0 = 0.3$. We varied α from $\{1.05, 1.1, 1.15, \cdots, 1.95, 2\}$ in steps of 0.05. In total, we simulated 400 experiments with different parameter values in which each experiment contained 700 trials. The data simulation procedure is summarized in Algorithm 1. For each set of fixed parameters (i.e. $\{v, a, zr\}$) an experiment with 700 trials is simulated with all α values. Thus for each parameter set we have $\{v, a, zr, \alpha_1, ACC_1, RT_1\} \cdots \{v, a, zr, \alpha_{20}, ACC_{20}, RT_{20}\}$. Then each parameter set is mapped to the correlation values of the $\alpha = \{1.05, 1.1, 1.15, \cdots, 1.95, 2\}$ with $Accuracy = \{ACC_1, \cdots, ACC_{20}\}$ and $MRT = \{mean(RT_1), \cdots, mean(RT_{20})\}$.

Algorithm 1 Lévy Flights Data Simulation

procedure GET CORRELATION OF RT AND ACC(Δt, t_0, v, zr, a)
  ACC, RT = [ ]
  for α in $\{1.05, 1.1, 1.15, \cdots, 1.95, 2\}$ **do**
    for i in $\{1, 2, \cdots, 700\}$ **do**
      $t \leftarrow 0$
      $X \leftarrow zr \times a$
      while $X < a$ and $X > 0$ **do**
        $e \leftarrow$ generate from $p^{\alpha}(x)$
        $X \leftarrow X + v \times \Delta t + e \times \Delta t^{\frac{1}{\alpha}}$
        $t \leftarrow t + \Delta t$
      end while
      Rt.append(t_0 + t)
      if $X \geq a$ **then**
        ACC.append(1)
      else
        ACC.append(0)
      end if
    end for
  end for
  return ACC, RT
end procedure

Simulation results

To get a clearer sense of how the α parameter relates to summary measures of performance (i.e., accuracy and RT) in different regions of the parameter space of the LF model, we plot the correlation between α and either accuracy or RT as a function of the best-fitting estimate of other core model parameters (e.g., mean drift rate, boundary separation, and start-point). Figure A.21 shows the pattern of correlations with RT and Figure A.22 shows correlations with accuracy in different regions of the parameter space. The effect of drift rate and threshold on the correlation between α and RT is demonstrated in Figure A.21a. It is clear that both the drift rate and threshold affect this correlation. When both the drift rate and the threshold have high values, the correlation tends to 1 and when both of them have low values the correlation tends to -1. This means when the threshold and the drift rate are high, response times increase as the prevalence of jumps in the accumulation process is reduced (i.e., $\alpha \longrightarrow 2$). In other words, when a cautious decision threshold with high drift rate is adopted, then a more stable accumulation process has a longer survival time. On the other hand, when the drift rate and threshold are low, a lower prevalence of jumps in evidence accumulation is also associated with faster RTs. This means when the threshold and the drift rate have low values, a more stable accumulation process finishes sooner.

Similarly, Figure A.21b presents the pattern of bias and threshold effects on the α-RT correlation. In this figure, the influence of threshold is clear—correlations between α and RT change from negative to positive as threshold increases—but at high values of the threshold, the effect is moderated by bias (i.e., by increasing the starting point bias value the correlation between α and RT decreases). In addition, Figure A.21c shows that there is no obvious pattern for the α-RT correlation in the bias and drift rate space. Finally, Figure A.21d represents that the α-RT correlation has symmetric behavior in the parameter space, producing a bimodal distribution of strong positive and negative correlations.

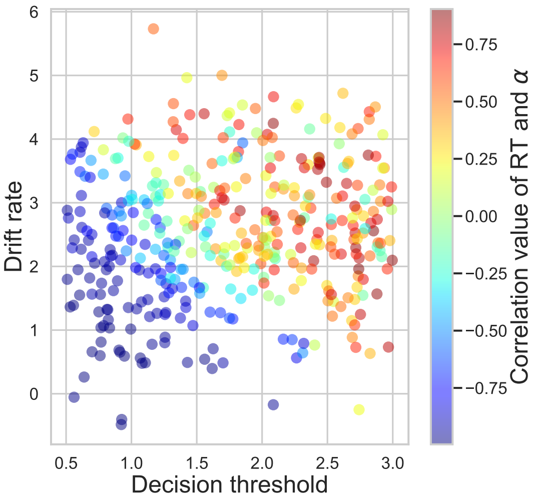

(a)

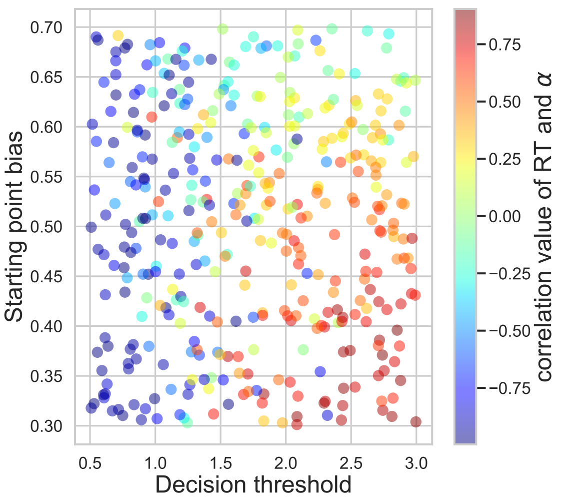

(b)

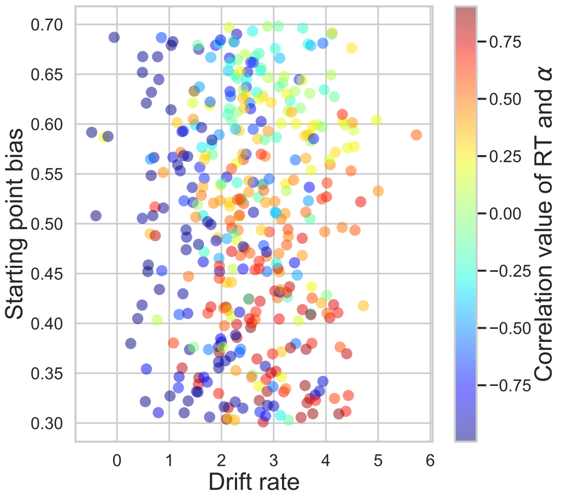

(c)

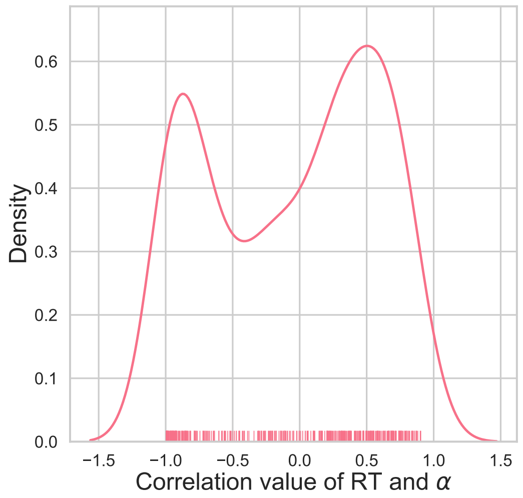

(d)

Figure A.21. Correlations between α and RT in different regions of the parameter space. (a) The pattern of α-RT correlation with respect to threshold and drift rate. (b) The pattern of α-RT correlation with respect to threshold and bias. (c) The pattern of α-RT correlation with respect to bias and drift rate. (d) Distribution of α-RT correlation in the parameter space. We refer the reader onto the online version of this article for the color version of this figure.

Figure A.22 illustrates α-ACC correlations. Unlike correlations with RT, α correlations with accuracy only appear to be affected by decision threshold, with no obvious systematic relationships with bias or drift rates. When thresholds are high, accuracy improves and is accompanied by reductions in jumps in the accumulation process (i.e., with a high decision threshold, a more stable accumulation process gives better accuracy). On the other hand, when the threshold is low, increases in accuracy are accompanied by increases in the prevalence of jumps in the accumulation process (i.e., with a low decision threshold, a less stable accumulation process gives better accuracy). Finally, Figure A.22d presents that in almost all parts of the parameter space, accuracy improves as jumps in evidence accumulation become less prevalent.

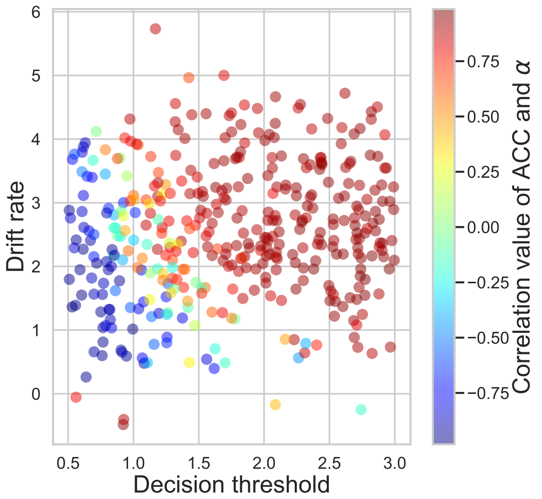

(a)

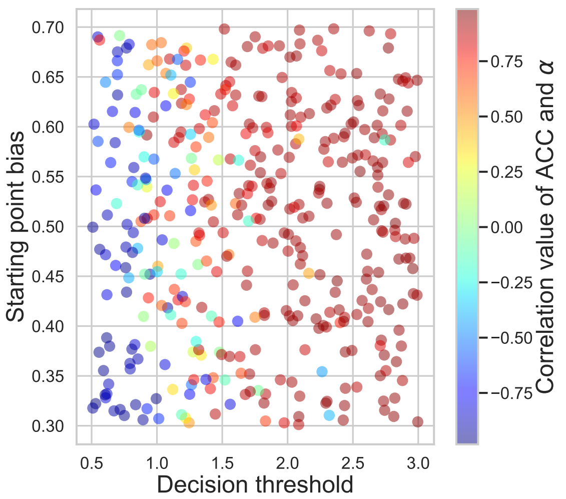

(b)

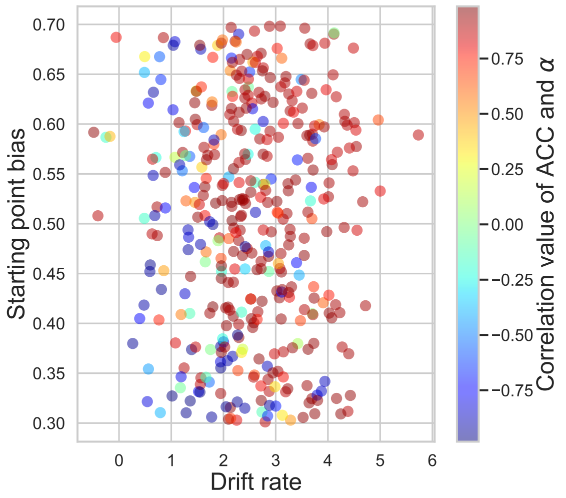

(c)

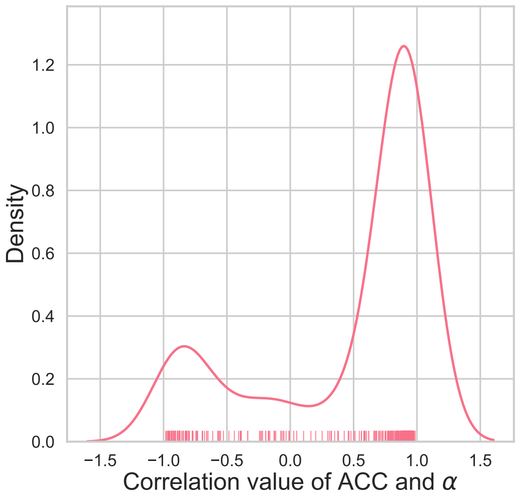

(d)

Figure A.22. Correlations between α and accuracy (ACC) in different regions of the parameter space. (a) The pattern of α-ACC correlation with respect to threshold and drift rate. (b) The pattern of α-ACC correlation with respect to threshold and bias. (c) The pattern of α-ACC correlation with respect to bias and drift rate. (d) Distribution of α-ACC correlation in the parameter space. We refer the reader onto the online version of this article for the color version of this figure.

Figure A.23 illustrates the pattern of correlation between α and the reward rate, defined as the proportion of correct responses divided by the mean correct response time. When the decision threshold is low (i.e., approximately less than 1) α correlates negatively with the reward rate (i.e., higher reward rates are achieved when jumps in evidence accumulation are more prevalent). On the other hand, when the decision threshold is high (i.e., approximately greater than 1) α correlates positively with reward rate (i.e., higher reward rates are achieved when jumps in evidence accumulation are less prevalent). While Figure A.23 shows a clear pattern for the impact of the threshold parameter on the correlation between α and the reward rate, we did not find any clear relationship with other parameters.

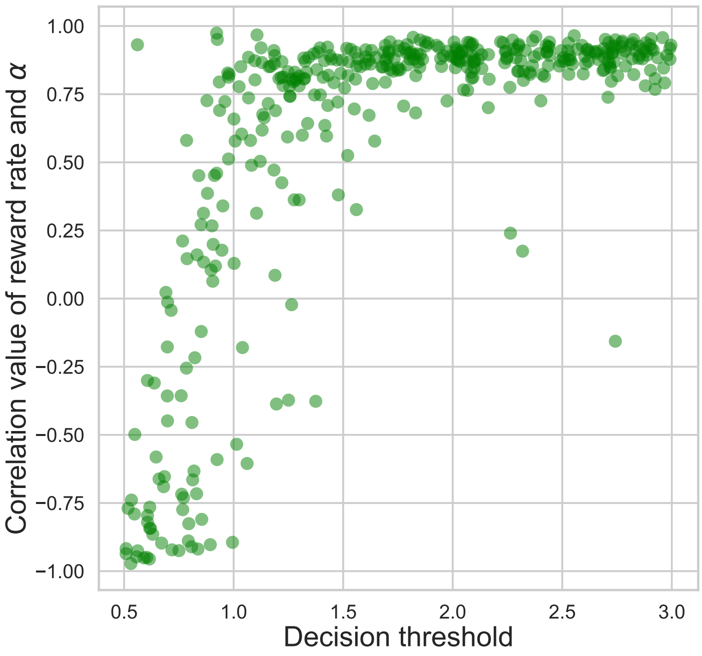

Figure A.23. Correlations between α and reward rate as a function of threshold.
